# An update on animal models of intervertebral disc degeneration and low back pain: Exploring the potential of artificial intelligence to improve research analysis and development of prospective therapeutics

**DOI:** 10.1002/jsp2.1230

**Published:** 2023-01-30

**Authors:** Mauro Alini, Ashish D. Diwan, W. Mark Erwin, Chirstopher B. Little, James Melrose

**Affiliations:** ^1^ AO Research Institute Davos Platz Switzerland; ^2^ Spine Service, Department of Orthopedic Surgery, St. George & Sutherland Campus, Clinical School University of New South Wales Sydney New South Wales Australia; ^3^ Department of Surgery University of Toronto Ontario Canada; ^4^ Raymond Purves Bone and Joint Research Laboratory Kolling Institute, Sydney University Faculty of Medicine and Health, Northern Sydney Area Health District, Royal North Shore Hospital St. Leonards New South Wales Australia; ^5^ Graduate School of Biomedical Engineering The University of New South Wales Sydney New South Wales Australia

**Keywords:** animal models of disc degeneration, artificial intelligence and deep machine learning, intervertebral disc, intervertebral disc degeneration, intervertebral disc regeneration, low back‐pain

## Abstract

Animal models have been invaluable in the identification of molecular events occurring in and contributing to intervertebral disc (IVD) degeneration and important therapeutic targets have been identified. Some outstanding animal models (murine, ovine, chondrodystrophoid canine) have been identified with their own strengths and weaknesses. The llama/alpaca, horse and kangaroo have emerged as new large species for IVD studies, and only time will tell if they will surpass the utility of existing models. The complexity of IVD degeneration poses difficulties in the selection of the most appropriate molecular target of many potential candidates, to focus on in the formulation of strategies to effect disc repair and regeneration. It may well be that many therapeutic objectives should be targeted simultaneously to effect a favorable outcome in human IVD degeneration. Use of animal models in isolation will not allow resolution of this complex issue and a paradigm shift and adoption of new methodologies is required to provide the next step forward in the determination of an effective repairative strategy for the IVD. AI has improved the accuracy and assessment of spinal imaging supporting clinical diagnostics and research efforts to better understand IVD degeneration and its treatment. Implementation of AI in the evaluation of histology data has improved the usefulness of a popular murine IVD model and could also be used in an ovine histopathological grading scheme that has been used to quantify degenerative IVD changes and stem cell mediated regeneration. These models are also attractive candidates for the evaluation of novel anti‐oxidant compounds that counter inflammatory conditions in degenerate IVDs and promote IVD regeneration. Some of these compounds also have pain‐relieving properties. AI has facilitated development of facial recognition pain assessment in animal IVD models offering the possibility of correlating the potential pain alleviating properties of some of these compounds with IVD regeneration.

## Graphical abstract

Animal models of IVD deneration have yielded invaluable information on the pathobiology of this degenerative condition and identified prospective therapeutic targets.The complexity of the degenerative changes and multiple therapeutic targets identified by these models suggests artificial intelligence methodology may be required to unravel these complexities and provide a rationale way forward in the development of effective repair strategies.

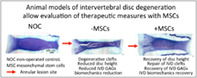

## 1 INTRODUCTION

The present review was undertaken to provide an update of the highly cited publication by Alini and colleagues: *Are animal models useful for studying human disc disorders/degeneration* published in 2008 in The European Spine Journal.^1^ This paper with >750 citations (Google Scholar August 2022) illustrates the importance and high level of interest in animal models of intervertebral‐disc degeneration (IVDD). Animal models have been invaluable in increasing our understanding of disc biology, however because of differences between species, care is required in the formulation of specific questions and selection of appropriate animal models if the intention is to validly extrapolate findings to human targets and therapeutically target IVDD in patients. In line with this, the previous review advocated that much more effort was needed to undertake research on human disc material and administrators/legislators were requested to improve access to human tissues for research purposes. Notwithstanding the need for human‐animal comparative studies and validation of model translational utility, there has been a veritable explosion of data in the area of IVDD pathobiology. While recognizing the potential posturally related biomechanical advantage, the use of primate and bipedal mouse or rat models are considered inappropriate on ethical grounds and the previous review recommended that these models should be discontinued. Studies however have continued to be published using bipedal rodent models particularly in scoliosis studies (Table 1),^2^, ^3^, ^4^, ^5^, ^6^, ^7^, ^8^, ^9^, ^10^, ^11^ with limited new information generated applicable to human IVDD.

**TABLE 1:** Studies using bipedal rodent spinal models

Model	Features	References
Bipedal rat scoliosis model	Melatonin antagonist (Luzindole) and improvement in the treatment of the bipedal scoliotic rat	7
Bipedal mouse scoliosis model	Enhanced central leptin activity in a scoliosis model in bipedal mice	5
Bipedal mouse scoliosis model	Assessment of whether elevated OPN has a role in the development of scoliosis in bipedal mice	6
Bipedal rat/mouse scoliosis models	Animal models of scoliosis	2
Bipedal mouse model	The effect of sympathectomy on the development and progression of scoliosis in bipedal mice	9
Bipedal mouse model	Radiologic and histomorphometric study on a bipedal C57Bl6 mouse model. Modulation of estrogen receptor expression prevents scoliotic curve progression	3
Rat bipedal kyphoscoliosis model	A model of kyphoscoliosis created by a scapula‐to‐contralateral ilium tethering procedure in bipedal rats	4
Bipedal standing mouse model	Novel bipedal standing mouse model of intervertebral disc and facet joint degeneration	11
Bipedal rat model	Effect of axial vertical vibration on degeneration of lumbar intervertebral discs	10
Bipedal mouse model of IVD and facet joint degeneration	Development and characterization of a novel bipedal standing mouse model of intervertebral disc and facet joint degeneration	8

Since the prior review three new large animal species (alpaca/llama, equine and kangaroo) have been advocated for IVD studies^12^, ^13^, ^14^, ^15^, ^16^, ^17^, ^18^, ^19^, ^20^, ^21^, ^22^, ^23^, ^24^, ^25^, ^26^ ongoing studies will determine how useful these new models actually are and whether they offer advantages or novel insights compared with existing more widely explored species. A major aim of using large animal models is to produce data that can be informative to comparative size and biomechanical events occurring in human IVD. Beyond these considerations however, animal IVDs to be analyzed should also approximate human IVDs in terms of structure and the cell types they contain. This immediately indicates some limitations with animals that have been used for IVD studies which unlike humans contain abundant notochordal cell populations and gelatinous NPs late into IVD maturation, for example, pigs and non‐chondrodystrophoid (ChD) dog breeds.^27^, ^28^, ^29^ In 2008, it was advocated that the non‐ChD‐canines were unsuitable for the development of a translationally relevant animal models of human IVDD.^1^ Non‐ChD‐canine breeds show how notochordal cells delay IVDD and regulate resident disc cell populations to improve their regenerative properties (see detailed discussion later in this review). With the completion of the canine genome (see Appendix S1) and establishment of differences between canine breeds, it is now abundantly clear that ChD‐breeds should be used to study degeneration of the IVD.^30^, ^31^ Canines display more phenotypic variation than any other mammals and are affected by a wide variety of diseases of a genetic origin.^32^, ^33^, ^34^ This reinforces that while mixed breed dogs may be more accessible for research purposes, canine breeds of a well‐defined pedigree should be used for spinal studies (see supplemental information). Development of the current day domestic dog represents a dramatic unprecedented long‐term evolutionary experiment on a large wolf‐like progenitor, with unparalleled phenotypic diversity.^35^

Animal genetics also need to be considered when selecting sheep breeds for IVD studies (see Appendix S1). The pedigree Iberian Merino was first transported to South Africa then Australia by General Macarthur in 1797 with The First Fleet. This was a relatively small animal compared with the modern day Merino but like its ChD‐canine counterpart, reproduced many aspects of the degenerative pathology of human IVDs including ingrowth of blood vessels and nerves into annular lesions,^36^, ^37^ remodeling of vertebral bone,^38^ osteoarthritic changes in facet joints,^39^, ^40^ degenerative changes in end‐plate vascularization,^41^ generation of radial and concentric tears in the IVD^42^ and consequential changes in IVD composition and biomechanical performance.^43^

## 2 THE INVOLVEMENT OF SEX HORMONES IN INTERVERTEBRAL DISC PATHOBIOLOGY

An aspect that has emerged in the last decade relevant to the development of prospective animal models of IVDD is the role that sex hormones may have. Although the effects of sex hormones on the metabolism of IVD cells was first identified in 1969^44^ it is only in the last decade that these have been shown to significantly impact on degenerative processes in the IVD.^44^, ^45^, ^46^, ^47^, ^48^, ^49^, ^50^, ^51^, ^52^, ^53^, ^54^, ^55^, ^56^, ^57^, ^58^, ^59^, ^60^, ^61^, ^62^ It is therefore critical that both male and female animals be examined and data collected and separately analyzed to ensure any pathophysiological sexual dimorphism is identified. Ideally this would include both actively cycling and “post‐menopausal” (likely gonadectomized) female cohorts to mimic and model clinically relevant human populations. Male sex hormones also effect IVD cells but age‐related decline in these does not occur to the same degree as females, suggesting that unless there is a specific research question, routine inclusion of gonadectomized male cohorts may not be ethically justified from a translational perspective.^46^

Low back pain (LBP) is a common symptom of premenstrual syndrome (PMS), experienced by most women during menstruation and may be exacerbated by premenstrual dysphoric disorder and dysmenorrhea or may be a symptom of endometriosis.^63^, ^64^ Female sex hormones play an important role in the etiology and pathophysiology of a number of musculoskeletal degenerative diseases, around 70% of perimenopausal women will experience LBP symptoms due to estrogen deficiency and estrogen decrease may be a risk factor for lumbar disc degeneration.^65^, ^66^, ^67^, ^68^, ^69^, ^70^ Postmenopausal women show accelerated IVDD due to relative estrogen deficiency, increased prevalence of spondylolisthesis, and facet joint osteoarthritis, in the first 15 years post menopause.^46^ Continued progression of lumbar disc degeneration in postmenopausal women has been observed.^46^ Further studies with functional foods and neutraceutical supplements under evaluation for their abilities to alleviate pain may prove to be a useful non‐drug treatment for post‐menopausal back pain.^71^

Estrogen can prevent the development of IVDD through its anti‐apoptotic properties inhibiting the production of the inflammatory cytokines IL‐1β and TNF‐α by disc cells.^72^, ^73^ This reduces catabolic events in the IVD by preventing the up‐regulation of MMPs induced by these inflammatory mediators. Estrogen also induces anabolic processes in the IVD by activating the PI3K/Akt pathway and also decreases oxidative damage.^74^ By inhibiting IVDD, estrogen exerts protective effects that prevent degradative structural changes in the IVD that would otherwise pre‐dispose the IVD to nerve ingrowth and production of neurotrophic factors and inflammatory mediators by IVD cells that contribute to IVD nociceptor activation and mechano‐sensitization of disc afferent nerve fibers leading to the generation of LBP.^75^, ^76^ A model of IVDD developed in rats has shown that inflammatory mediators and neurotrophic factors secreted by IVD cells in the degenerate IVD produces an environment in this tissue that promotes nerve ingrowth^77^ and the sensitization of mechanoreceptors and nociceptors by dynamic compression of the IVD.^78^ Dynamic compression of the degenerate IVD produces long‐lasting increases in IVD inflammatory mediators and nociceptor activation leading to the generation of LBP.^78^

## 3 SMALL ANIMAL MODELS OF IVDD

Despite the high disability rates of LBP and high socioeconomic impact in adult humans, the discovery of new drug treatments for the alleviation of chronic mechanical LBP is lacking due to a paucity of knowledge of LBP pathobiology. The use of animal models of LBP aims to alleviate this deficiency. Compared with large animal models of IVDD the husbandry and handling of mice is straight forward and their relatively short generational period and ability to manipulate murine genes has made these a popular and very useful animal model. This is reflected in the large number of mouse studies which have been conducted in the last decade to elucidate the multifactorial components that contribute to IVDD.^79^

The bipedal rat and mouse models continue to be used despite ethical issues expressed in our review of 2008 (Table 1). Quadruped rodent models have also continued to be a popular animal model for IVD studies. The sheer numbers of rodent spinal studies that have appeared in the last decade points to their accessibility and utility through genetic manipulation. Whether this popularity equates with translational relevance and value remains to be determined. Studies using rodent IVD models outnumber any other animal model in the last decade. The mouse is a well‐established medical‐research experimental model, genes can be easily found in the mouse genome sequence, and it is also possible to test experimentally the function of those genes and to test possible therapeutic agents and evaluate their precise effects. The Mouse ENCODE (ENCyclopedia Of DNA Elements) project is building a comprehensive catalogue of functional elements in the mouse genome, comparing these to the human.^80^, ^81^ These include protein and non‐protein coding genes and selection elements that regulate which genes are turned on or off, and when this happens in development. Mice and humans share about 97.5 per cent of their working DNA; on average, the protein‐coding regions of the mouse and human genomes are 85 percent identical; some genes are 99 percent identical while others may share only 60 percent identity. The mouse genome was published in 2002 and found to be comparable to the human genome in terms of its size and the genes it encompassed.^82^ This has enabled the development of thousands of mouse strains with mutations mirroring those seen in human genetic diseases.^83^ These models have been an invaluable resource in determining the potential roles of specific genes in human diseases.^79^

An extensive review on the mouse IVD was published in 2021^79^ and in order to avoid duplication, the interested reader is referred to this review for specific information on models of the mouse IVD. Some supplemental information on the mouse is also provided at the end of this review (see supplemental information).

## 4 GENETIC STUDIES USING ANIMAL MODELS

## 5 CANINE AND OVINE GENOMIC RESEARCH

Considerable progress has been made in the elucidation of the canine genome,^30^, ^84^ this highlights canine breeds which evolved from the ancestral wolf genome and the susceptibility of different canine breeds to specific diseases^85^, ^86^, ^87^, ^88^, ^89^, ^90^, ^91^, ^92^ (see Appendix S1).

**FIGURE 1:**
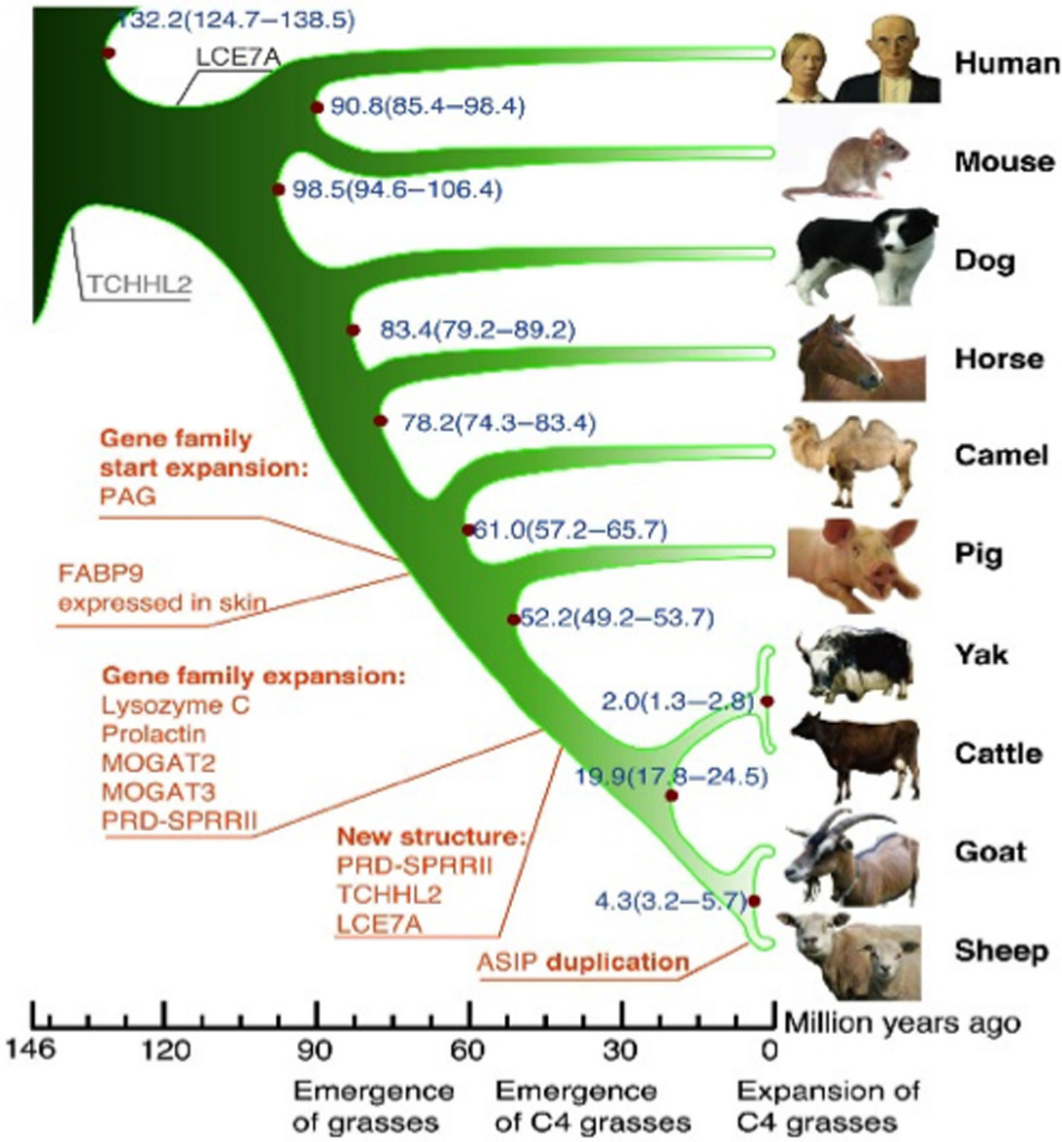
Phylogenetic tree of sheep and related animals. generated using single‐copy orthologous genes. Origins (black) and amplificationgenes (red). Scale is in millions of years ago (Mya). Blue numbers on the nodes are calculated divergence times from present (Mya) and its confidence interval. Figure reproduced from^93^ under Open Access

The ovine genome has also been published by The International Sheep Genomics Sequencing Consortium and illustrates the complexities of a four stomach ruminant animal and its evolution compared with other mammals^93^ (Figure 1; Appendix S1).

## 6 NEW LARGE ANIMAL MODELS OF IVDD

**FIGURE 2:**
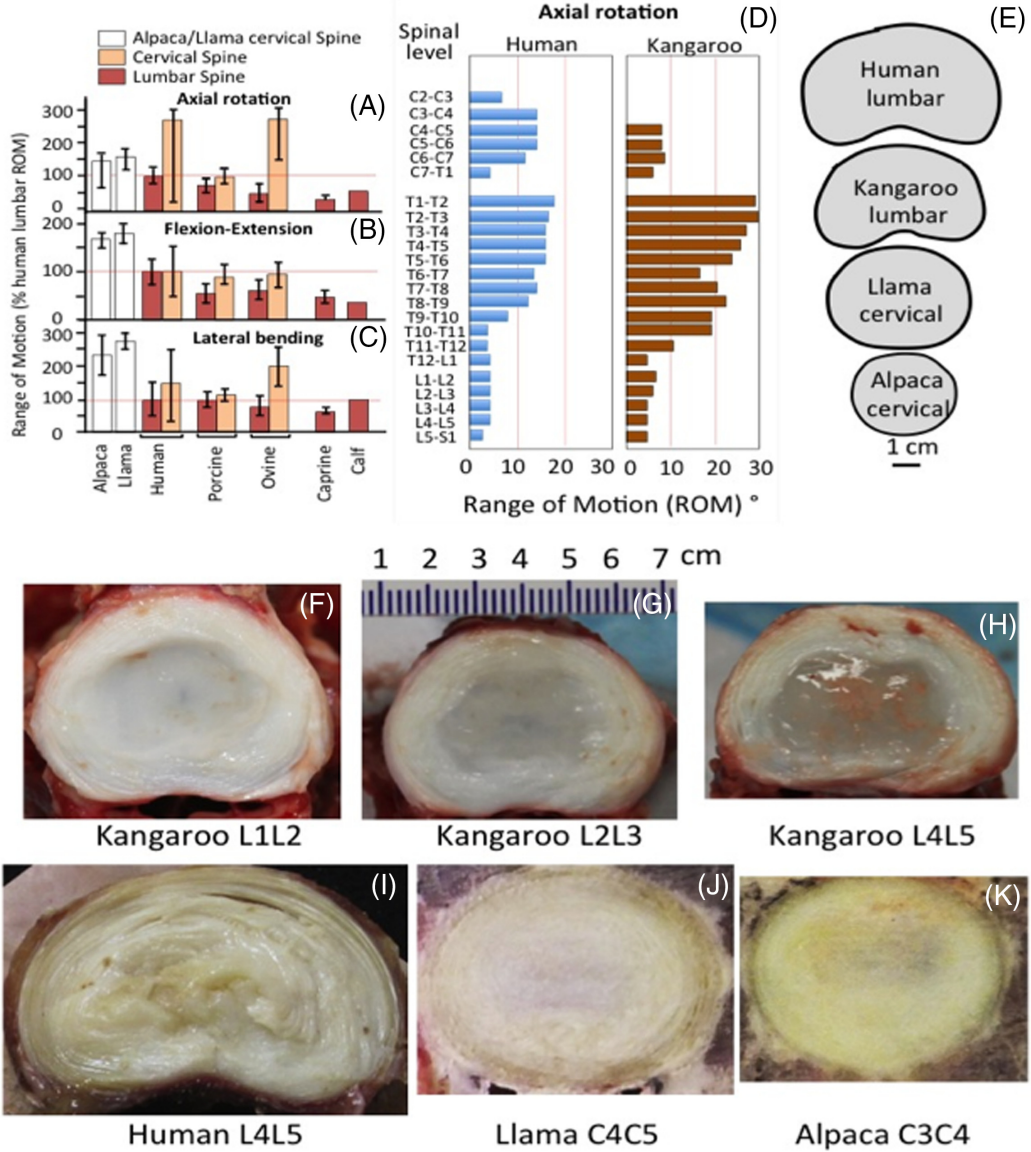
Approximate range of motion (ROM) (A‐C) of animal cervical and lumbar spine segments in axial rotation (A) flexion‐extension, (B) lateral‐bending (C) as a percentage of human lumbar ROM and axial rotation at different spinal levels for the kangaroo and humans (D).^15^, ^97^, ^98^, ^99^, ^100^, ^101^ References for benchmark data are: Human Lumbar,^101^, ^102^ Porcine Lumbar and Porcine Cervical,^98^ Ovine Lumbar and Ovine Cervical,^99^ Caprine Lumbar,^15^ Calf Lumbar.^103^ Relative sizes of human and kangaroo lumbar and cervical Llama and Alpaca IVDs (E). Macroscopic views of horizontally bisected kangaroo L1L2 (F), L2L3 (G) L4L5 (H), human L4L5 (I) and Llama C4C5 (J) and Alpaca C3C4 (K) IVDs. Images F‐H were kindly supplied by Dr Uphar Chamoli, Director of Engineering, Kunovus Pty Ltd, School of Biomedical Engineering, University of Technology, and St. George Clinical School, University of NSW, Sydney, Australia. Images J, K reproduced from^21^ with permission.

Macropod, Equine and Cammelid species have been proposed for IVD studies and these represent interesting developments.^12^, ^13^, ^14^, ^15^, ^16^, ^17^, ^19^, ^20^, ^21^, ^22^, ^23^, ^24^, ^25^, ^26^, ^94^, ^95^, ^96^ The equine IVD is anatomically quite different from other species in having a ball and cup type structure. The kangaroo tail IVD is unique in being weight‐bearing and these and other macropdods are the only animals with five appendages.^18^ Although macropods assume a quadrupedal gate at very slow speeds, they are bipedal at higher speed and thus their spine is loaded vertically more akin to humans. The thoracolumbar alpaca disc has a similar cross‐sectional profile to bovine caudal IVDs (Figure 2) and is reported to undergo degenerative changes.^20^, ^21^ Further studies are required to determine if they contain notochordal cell populations in adulthood. It remains to be seen how cost effective these new models will be, availability issues may also be disincentives to their routine use as models of IVDD.

## 7 CAMMELIDS

Cammelids (Alpacas and Llamas) have large IVDs with some similarities to human spinal anatomy,^21^ size and biomechanical flexibility,^20^ and natural history of spontaneously occurring IVDD.^19^, ^25^ However, they have a dissimilar cross‐sectional area closer to that of bovine caudal IVDs.^20^, ^21^ The prevalence of age‐dependant IVDD in the alpaca cervical spine^21^ suggests it may be worthy of further examination. Compressive myelopathy due to IVD herniation in a llama (*Lama glama*) has been reported.^25^ It is difficult to envisage the widespread use of the cammelids as models of disc degeneration given that they are not widely available, are expensive and have more difficult husbandry (e.g., fencing requirements). The cervical cammelid IVD is significantly more flexible than human IVDs displaying higher ROM in flexion extension and lateral bending.^21^

## 8 EQUINE SPINAL STUDIES

**FIGURE 3:**
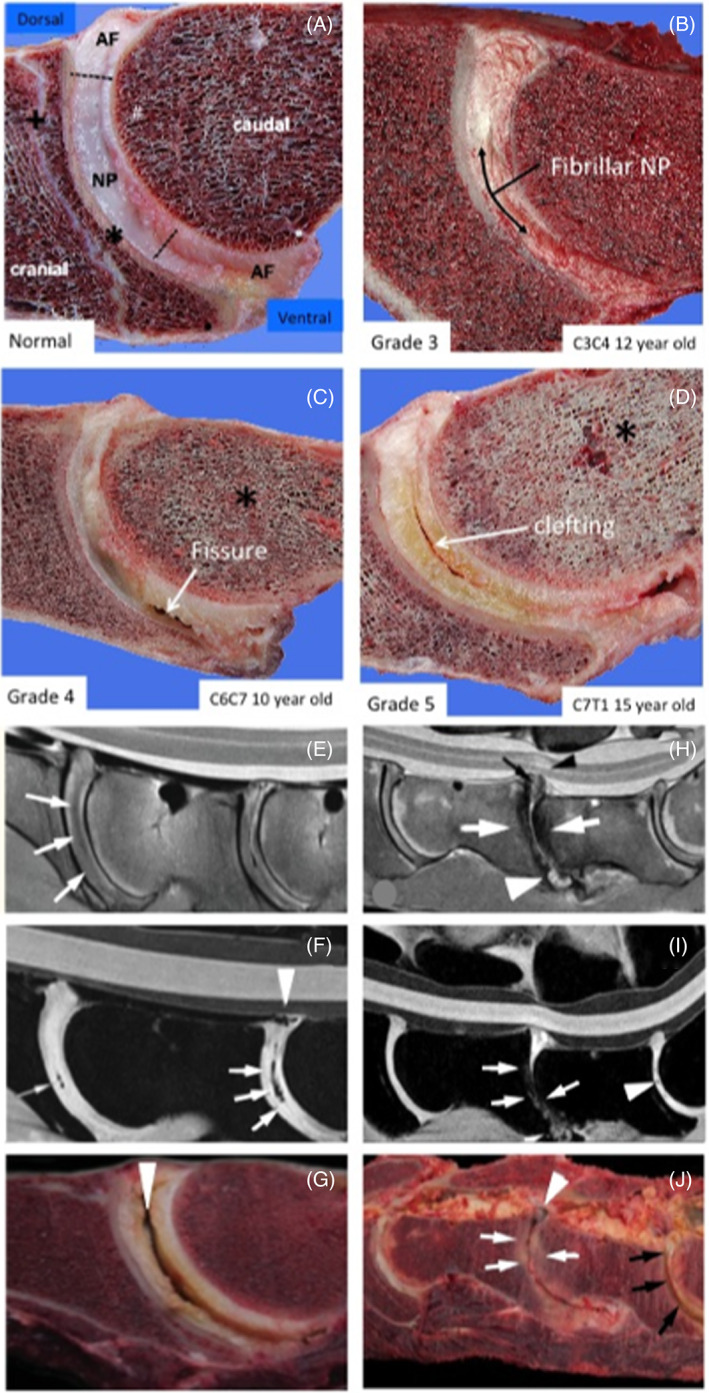
Vertical sections through equine spinal segments showing the characteristic ball and socket appearance of an equine IVD and characteristic tears and lesions that occur with IVDD (A–D). MRI images of equine spinal segments showing reduced MRI signal in the IVD with the onset of IVDD (E, H, F, I) correlating with lesions evident on gross examination of spinal segments (G, J). IVD features are annotated and degenerative clefts and fissures indicated with arrows and decreased bone density changes with an asterisk in (A–D). Images A–D reproduced from^13^ under Open Access Attribution‐noncommercial 4.0 International (CC BY‐NC 4.0) license. Diffuse hypo intense regions and signal voids and diffuse hypointense areas are noted throughout the intervertebral discs with loss of definition of the nucleus (white arrows); B, sagittal water selective cartilage image; noted clefts (white arrows) and regions of the dorsal longitudinal ligament of C6‐C7 (white arrow head); and diffuse hypointense areas were noted throughout the IVDs with loss of definition of the nucleus (white arrows); Clefts and loss of NP definition are indicated with white arrows and defects in the longitudinal ligament attachments with arrow heads (E–J). A large central cleft in a C6C7 IVD is indicated by an arrowhead in (G). Severe remodeling of CEPs and discolouration of protruding dorsal IVDs (white arrows) in the C6C7 IVD (J). The C7S1 IVD displays severe cleft formation and yellow discolouration (black arrows)

The normal and degenerate equine spine has undergone radiographic and MRI analyses.^22^, ^94^, ^95^ Biochemical and biomechanical studies have also examined cervical facet joint cartilage,^96^ and IVD tissues^13^, ^24^ in normal IVDs and those which have undergone prolapse and diskospondylitis.^12^, ^17^ The morphology and mobility of normal and prolapsed equine IVDs have also been examined.^16^, ^23^, ^26^ These thoracic and cervical IVDs have characteristic concave and convex cartilaginous endplates (Figure 3). Equine IVDs appear more like a fibrous articulating “ball and cup joint” rather than a typical IVD as seen in other animals. The equine IVD nevertheless displays degenerative tears and lesions consistent with a major weight bearing structure.

## 9 KANGAROO

Kangaroo and human lumbar IVDs have similar flexibility profiles.^14^ Similarities in thoracic spinal vertebral geometry in the vertebrae, pedicles and facet joints, makes the kangaroo a clinically relevant human surrogate for testing spinal implants. The thoracic kangaroo spine however is significantly more flexible than the human (Figure 2d). The kangaroo is a pseudo‐biped (at higher ambulatory speeds) but it also the only animal with “5 load‐bearing limbs”, the tail IVDs contribute to spinal weight bearing when walking. Forces through the pelvis to the lumbar IVDs in the kangaroo differ when the tail is stabilizing the spine through the pelvis. The juncture of the mobile lumbar IVDs with immobile pelvis is a major determinant of the metabolism of the disc cell populations in human lumbar IVDs contributing to the higher incidence of disc degeneration at the L4L5 as well as the lumbosacral intervertebral disc. The kangaroo's tail acts as a counterbalance to the body during hopping^18^ but has complementary roles during walking where it is used as a muscular appendage and additional leg to support, propel and power their motion. The muscles supporting the vertebrae and tail IVDs in the kangaroo are very rich in mitochondria indicating they have important roles in the energetics of this very capable support structure. The tail is planted on the ground when the kangaroo is in a walking mode and their front and hind legs assume a distinctive gait referred to as “pentapedal” locomotion,^14^, ^104^, ^105^ with the fifth point of contact being the tail. Kangaroo tails contain more than 20 vertebrae and adjacent IVDs and are biomechanically and physiologically capable structures.^18^ The dense tail muscles are much larger than the muscles of the front limbs and their abundant mitochondria indicate they have a large aerobic capacity.^106^ Studies of the structure of the tail IVDs of the kangaroo have yet to be undertaken, it would be interesting to ascertain how similar they were to bovine caudal IVDs which some researchers have shown to be useful investigative tissues. However, since kangaroo tail IVDs are weight bearing they may be even more relevant for such studies, as it is a uniquely adapted axial support structure.^18^, ^107^, ^108^

## 10 THE CANINE MODEL OF IVDD

A number of studies have been conducted demonstrating the applicability of the ChD‐canine as a model of IVDD (Table 2). A number of therapeutic agents have been evaluated using these models including mesenchymal stem cells and a number of bioscaffolds and hydrogels employed using a number of tissue engineering approaches.

**TABLE 2:** The canine as a model of IVDD

Model	Features	References
ChD canine	A clinically relevant intermediate‐sized animal model of IVD‐associated spinal pain	109
ChD and non‐ChD canine IVD	Cervical IVD herniation in ChD and non‐ChD small breed dogs	110
Canine	IVDD in dogs: consequences, diagnosis, treatment.	111
Canine	Canine IVDD model	28, 112
Canine	Comparison of magnetic resonance imaging, and histological findings in surgically treated IVDs	113
Canine	Incidence of IVDD‐related diseases and associated mortality rates	114
Canine	Canine model of IVDD	27
Canine	Macroscopic grading of IVDD degeneration using the Thompson system and comparison with low‐field MRI	115
ChD and non‐ChD canine IVD	Evaluation of IVDD in dogs by use of Pfirrmann grading and low‐field magnetic resonance imaging	116
Canine	A review of IVDD in dogs	117
Canine	Retrospective study of IVD herniation in dogs in Japan	118
Canine thoracolumbar IVD	Canine thoracolumbar IVDD: diagnosis, prognosis, and treatment	119
ChD and non‐ChD canine IVD	A histopathological comparison of intervertebral disc degeneration in chondrodystrophic and non‐chondrodystrophic dogs	120

Therapeutic agents examined for canine disc regeneration	
Procedure	References
Human bone morphogenetic protein 7 transfected NP cells delay the degeneration of the canine IVD	121
Effects of osteogenic protein‐1 on intervertebral disc regeneration in animal models	122
Intradiscal application of rhBMP‐7 does not induce regeneration in a canine model of spontaneous IVDD	123
NP cells expressing hBMP7 can prevent the degeneration of allogenic IVD in a canine transplantation model	124
NTG‐101 molecular therapy halts progression of canine DDD	125, 126
Restoration of IVD integrity by intradiscal delivery of Celecoxib‐loaded microspheres	127
IVD repair using Link‐N	128
Extradural morphine use in postoperative analgesia in dogs undergoing IVD surgery	129

Use of MSCs, notochordal cells/notochordal conditioned media in regenerative procedures on the canine IVD	
Procedure	References
Treatment of naturally degenerated canine lumbosacral IVDs with autologous MSCs	130, 131
Use of notochordal cell‐derived matrix for biological repair of the IVD	132
Therapeutic use of bone marrow MSCs to treat canine IVDD	130, 131
Notochordal‐cell derived extracellular vesicles exert regenerative effects on canine and human NP cells	133
Treatment of canine IVDD using notochordal rich NP	126
BMP‐2, but not MSCs, exert regenerative effects on Canine and Human NP cells	134
A review of MSC and bioscaffold use in disc repair and regeneration	135
A review of the use of MSCs in the treatment of IVDD in companion animals	136
Transplantation of adipose derived MSCs for acute thoracolumbar disc disease	137
Human Wharton's jelly cell transplantation for the treatment of canine IVDD	138
Coculture of notochordal, NP and MSCs for canine IVD regeneration	139
Notochord conditioned medium stimulates ECM production by NP and MSCs	140
Regenerative treatment strategies for IVDD in dogs	141
Canine notochordal cell‐secreted factors protect murine and human NP cells from apoptosis by inhibition of activated caspase‐9 and caspase‐3/7.	142
IVD‐derived stem cells: implications for regenerative medicine and neural repair	143
Cell number effects on MSC transplantation in canine IVDD	144
MSCs arrest IVDD through chondrocytic differentiation and resident IVD cell stimulation	145
Cell transplantation for the treatment of lumbar IVDD	146
MSCs for the treatment of canine IVDD	147

Bioscaffolds evaluated for the repair of the canine IVD		
Scaffold	Functional properties	References
Thermoreversible HA‐ poly(*N*‐isopropylacrylamide) (HA‐pNIPAM) hydrogels	Injectable thermoreversible HA‐hydrogel NP cell microbeads. Suitable for NP repair as an injectable carrier, maintained cell phenotype, ECM production.	148
Implantation of bullet fragments made from copper, lead, and aluminum alloys in lumbar IVDs	Impact of surgically implanted copper, lead, and aluminum alloy bullets in lumbar IVDs. Significant IVD tissue reactions noted with copper and lead. Copper produced severe IVDD aluminum alloy did not.	149
Poly(ε‐caprolactone‐co‐lactide)‐β‐poly(ethylene glycol)‐β poly(ε‐caprolactone‐co‐lactide) PCLA‐PEG‐PCLA hydrogel slow release celecoxib	Intradiscal application of a PCLA‐PEG‐PCLA celecoxib loaded hydrogel for the treatment of LBP. LBP in 9 of 10 dogs, evident by clinical examination and owner questionnaires.	127
Total disc replacement	Tissue‐engineered (TE) canine cervical IVDs from adult canine AF and NP cells seeded in collagen and alginate hydrogel IVD molds. At 4 and 16‐weeks NP cells surrounded by PG‐rich ECM, fibrocartilaginous ECM in AF. IVDs stable, stable disc height, hydration over 16 weeks and integration with host tissue.	150
Polyetheretherketone (PEEK)	Anterior canine cervical IVD fusion using bioactive polyetheretherketone. Bony fusion rate of TiO2‐coated PEEK ~40% by μCT, better fusion rates and bone‐bonding ability than uncoated PEEK implant.	151
Injection of polyester amide microspheres	Safety and biocompatibility of intradiscal injection of polyester amide (PEA) microspheres in a canine model of IVDD. Good cyto‐ and biocompatibility, safe sustained release of intradiscal delivery of biologics.	152
Injection of gelified ethanol into lumbosacral IVDs of healthy dogs	Short and long term effects of gelified ethanol in lumbosacral IVDs to treat IVD protrusions in humans via NP injection.	153
PEG and methylprednisolone sodium succinate (MPSS) in the treatment of herniated IVDs	Placebo‐controlled, prospective, randomized clinical trial of polyethylene glycol and methylprednisolone sodium succinate (MPSS) in the treatment of herniated IVDs, 47.6% dogs recovered ambulation	154
Celecoxib‐loaded poly‐N‐isopropylacrylamide hydrogel	Intradiscal application of a thermoreversible celecoxib‐loaded poly‐N‐isopropylacrylamide MgFe‐layered double hydroxide hydrogel	155
MSC loaded injectable microcryogel reinforced alginate	Microcryogel reinforced alginate encapsulation of MSCs for leak‐proof delivery alleviated canine IVDD	156
TE IVD allograft	Tissue‐engineered allograft IVD transplantation	157
NP hydrogel prosthesis: canine IVD.	Evaluation of a hydrogel NP prosthesis ex vivo	158
TE IVD composite	Evaluation of a TE IVD composite for disc regeneration	159
Alginate/chitosan hybrid scaffold	Alginate/chitosan hybrid fiber scaffold for AF cells	160

## 11 THE OVINE MODEL OF IVDD AND REGENERATION

**FIGURE 4:**
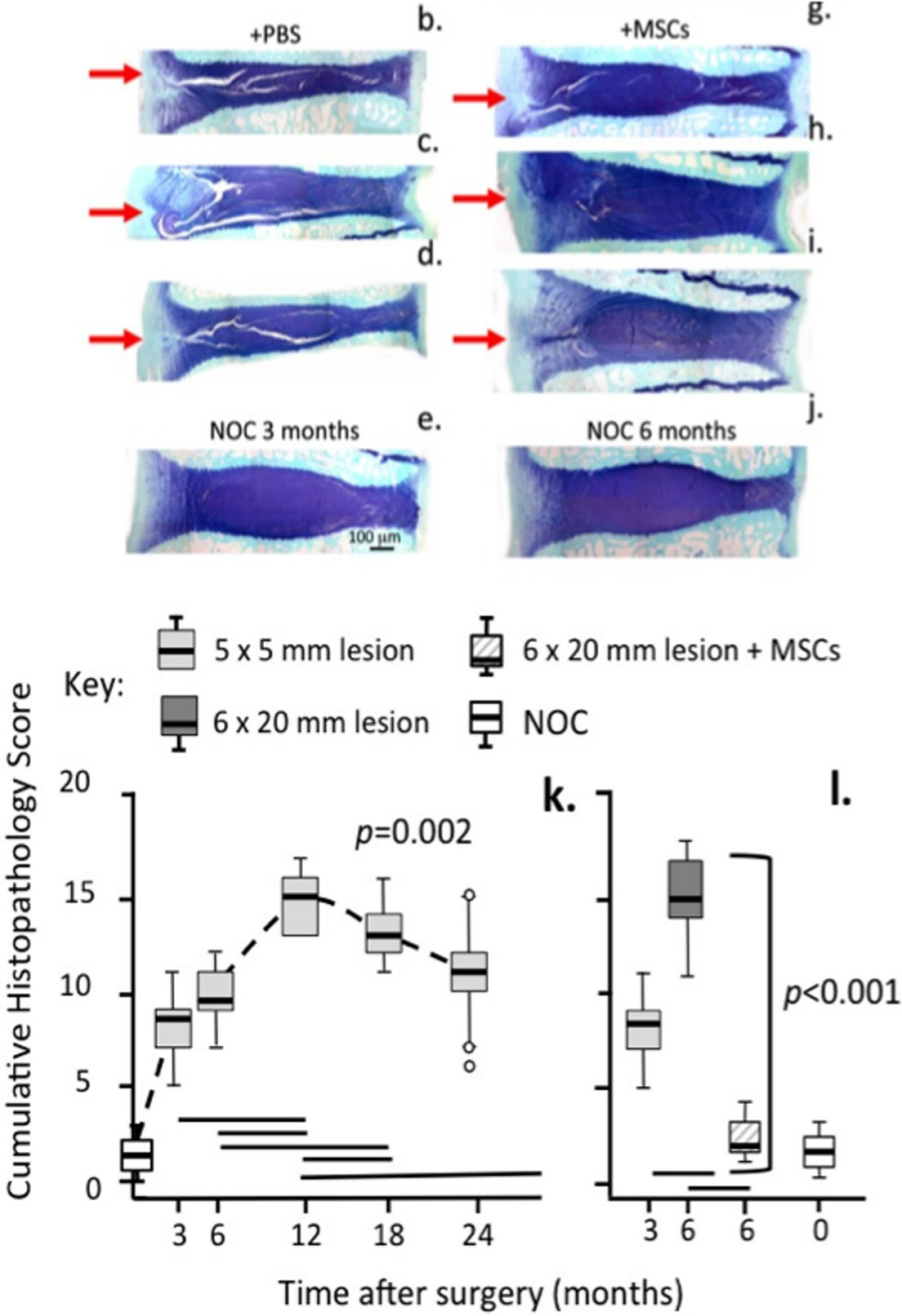
Depiction of how a large 6 × 20 mm outer anterolateral annular lesion (red arrow) destabilizes the ovine IVD and induces disc degeneration with propagation of the lesion into the IVD (b, c, d) and demonstration of the utility of bone marrow derived stromal stem cells for the regeneration of lesion affected IVDs (g, h, i). Toluidine blue‐fast green stained vertical IVD sections. Non‐operated control IVDs (e, j), freshly made lesion in a cadaver IVD (f). Disc degeneration was induced for 4 (b, c, g, h) or 12 weeks (d, i) then injected with PBS (b, c, d) carrier or MSCs (g, h, i) and recovery allowed to proceed for 8 (g, h) or 22 weeks (i). Annular lesions severely reduced the disc heights in the degenerate IVDs (b, c, d) but disc heights were recovered to ~95% of normal values in MSC treated IVDs (g, h, i) where repair of the IVD lesion occurred. In the corresponding PBS carrier treated discs (b, c, d) lesion development was extensive. Histopathological scoring of IVDs^179^ in which degeneration had been induced by a 5 × 5 (k) or 6 × 20 mm lesion (l) based on IVD structure, proteoglycan content, disc height, lesion progression, cellular infiltration showed a steady increase in the cumulative degenerate histopathology index in the 5 × 5 mm lesion over 24 months (k) and development of a similar histopathology score by 6 months in the 6 × 20 mm lesion (l) however the administration of MSCs reduced this degeneracy index to levels similar to those evident in non‐operated control IVDs. Figure modified by Open Access under an Attribution 4.0 International CC BY 4.0 license from^162^, ^179^

Of the canine, goat, ovine models the latter stands out as being particularly significant in terms of its similarity in structure to human IVDs and the spectrum of IVD degenerative pathobiological features reproduced in ovine models of experimental IVDD (Table 3). The large annular lesion mechanical destabilizing ovine model of experimental disc degeneration is an aggressive model utilizing a large 6 mm deep and 20 mm wide outer annular lesion.^161^ This lesion is of such a size that it severely disrupts normal annular architecture and annular biomechanical properties destabilizing the entire disc but it does not result in prolapse of the nucleus pulposus through the annular defect. The resultant mechanical destabilization results in dramatic changes in the gene expression profiles of the resident disc cell populations. Expression of MMP‐1 and 13, and ADAMTS4 and ADAMTS5 are elevated in lesion affected IVDs. Increased expression of type I collagen and type II collagen accompany the elevated MMP expression patterns indicating that anabolic and catabolic gene expression occur hand in hand with IVDD. Initially the annular defect site is filled with granulation tissue and an influx of blood vessels and nerves into the inner margins of the AF is evident. This does not occur in control IVDs, where sparse blood vessels and nerves are confined to the outermost lamella even in aged IVDs. Blood vessels and nerves encroach further into the destabilized disc when the NP becomes depleted of space‐filling proteoglycan as the disc degenerate and display a reduced disc height. However by 6 months post‐treatment of the degenerate IVD with bone marrow derived stromal mesenchymal stem cells (BMMSCs) a replenishment in NP proteoglycans was evident and a significant recovery (95%+) of normal disc heights was achieved.^162^ Blood vessels and nerves regressed from the repaired annular defect site as NP proteoglycan levels began to rise with the defect granulation tissue eventually being replaced entirely by new annular lamellae of increased size by 6 months post operation.^37^ This annular repair process was replicated by administration of HA oligosaccharides to the defect site which is consistent with the depolymerisation of HA known to occur under the inflammatory conditions present during IVDD.^163^ Furthermore, testing of the mechanical properties of MSC repaired IVDs showed they had re‐attained similar biomechanics to age matched ovine control discs.^162^ Thus, the repair of large annular lesion affected discs was very significant and strong evidence of the potential efficacy of BMMSCs for repair of degenerate human IVDs. No other animal model of disc degeneration has used such a large defect to induce disc degeneration. The successful repair of such a massive 6 × 20 mm defect is particularly significant given that this defect is not stabilized by fixation thus internal disc micro‐movement occurring during normal body locomotion would be expected to inhibit re‐attachment of damaged surfaces to one another during the annular repair process. This positive response with BMMSCs is an outstanding achievement not replicated in any other animal model of disc degeneration. The mechanism that drives IVD repair is probably due to local release of growth factors by the administered stem cells and the direction of resident IVD cell populations to participate in tissue repair processes. There is no evidence that the administered stem cells become engrafted long‐term despite the detection of some residual viable stem cells 1 month after administration. It is difficult to envisage that the resident nutritive system in the IVD would be capable of maintaining the viability of such a large number of administered cells. A growth factor mediated repair process has also been proposed in canine IVD studies.^125^, ^126^, ^164^ Earlier claims in rodent IVD studies using needle punctures to induce IVDD and MSCs to repair the IVD^165^, ^166^ do not compare with the significance of the findings produced with the ovine model (Figure 4). The ovine model has been awarded nine ISSLS^36^, ^167^, ^168^, ^169^, ^170^, ^171^, ^172^, ^173^, ^174^ and two Grammer Prizes.^175^, ^176^ This ovine model reproduces many of the degenerative features which characterize human IVDD. A number of studies have been conducted with the ovine model to examine spatiotemporal changes in discal and paradiscal components such as the NP,^177^ CEPs,^41^ facet joints^40^ and vertebral bone that occur adjacent to and distant from the lesion site.^38^ When the degenerate IVD becomes depleted of aggrecan it becomes susceptible to the ingrowth of blood vessels and nerves,^37^ focal expression of fibroblast growth factor (FGF)‐2, TGF‐β1 and α‐smooth muscle cell actin^178^ by cell populations associated with annular remodeling and attempted repair of the lesion site. In 2003 The ISSLS Prize was awarded for a study on the quantitation of nerve ingrowth into the degenerate ovine IVD (Table 3).^36^

**TABLE 3:** The Ovine model of IVDD and regeneration

Model	Features	References
Percutaneous nucleotomy model of IVDD	Lumbar spine degeneration following injury via percutaneous minimally invasive partial nucleotomy	180
Cervical IVDD and Regeneration Model	Cervical IVDD and regeneration model using a ventral surgical approach	181
Lumbar IVDD Models	Lumbar IVDD Models for evaluation and development of novel regenerative therapies	182
Stepwise model of IVDD	Stepwise model of IVDD with intact AF to test regenerative strategies	183
IVDD failure induced by complex posture/loading rate	Model of IVD failure induced by concordant complex posture and loading rate on motion segment failure	184
Far‐lateral disc herniation model	Far‐lateral disc herniation induced by a percutaneous posterolateral approach	180
Herniation model of IVDD	Herniation model induced by compression, flexion and facet‐constrained shear	171, 175
IVDD model used to assess regeneration	Transpedicular surgical approach for regenerative strategies for repair of IVDD	185
Ovine IVDD model	Disc disruption induced by low frequency cyclic loading	186
Drill bit injury of IVDD	IVDD model induced by a lateral retroperitoneal drill bit injury	187
Histopathological grading scheme for IVDD/regeneration	Histopathological grading of IVDD and the therapeutic utility of adult MSCs for IVD Regeneration.	179
Posterolateral disc prolapse model	Posterolateral disc prolapse in flexion initiated by lateral inner annular failure:	188
Nucleotomy model	Nucleotomy model with intact AF to test the efficacy of IVD disc regenerative strategies	189
IVD regeneration model	An assessment of the transpedicular approach as an alternative route for intervertebral disc regeneration	190
IVDD induced by a lateral lesion	Lateral surgical lumbar IVDD model	191
Large annular lesion destabilization model of IVDD	Mechanical destabilization induced by controlled annular incision of IVD dysregulates MMP and anabolic genes	161
Nucleotomy model of IVDD	Partial nucleotomy ovine model of IVDD	192

Bioscaffolds used in Regenerative strategies for the ovine IVD model		
Model	Feature	References
Minimally invasive repair model of IVDD	Minimally invasive repair of IVDD using injectable polymethyl‐methacrylate and bovine collagen	193
TE 3D IVD Biocomposite	IVD engineering of bio‐mimetic biocomposite mimicking the AF lamellae structure	194
Electrospun polycaprolactone	Electrospun‐aligned microfibrous implant for Annulus fibrosus repair	195
High‐density collagen gel	In‐vivo AF repair using high‐density collagen gel	196
Cervical IVD prosthesis	Cervical IVD prosthesis based on the physiological curvature of endplate for cervical disc replacement	197
Polyglycolic acid/polyvinylidene fluoride annulus implant	Repair in AF defects using a bio‐integrative implant in an in‐vivo ovine model	198
HA hydrogel, ionically crosslinked metacrylated gellan gum hydrogels	In vivo biofunctional evaluation of hydrogels for disc regeneration	199
Hydrogel, suture, NP tissue replacement, biological glue combinations	Hydrogels for nucleus replacement with an intact; annulus incision repaired by suture and glue; annulus incision with removal and re‐implantation of nucleus tissue; and two different hydrogels repaired by suture and glue.	200
Resorbable PGA‐HA implants	Regeneration of NP tissue in an ovine IVDD model by cell‐free resorbable polymer scaffolds	201
Oxidized HA gelatin implant	An injectable NP implant restores compressive range of motion in the ovine disc	202
IVD transplant	Preparation of intact bovine tail IVDs for organ culture	203
Construct of collagen gels seeded with ovine AF cells	Self‐assembly of aligned tissue‐engineered AF and IVD composite via collagen gel contraction	204

MSCs for repair of IVDD evaluated using the ovine model		
Model	Features	References
Large destabilizing lesion model of experimental IVDD	MSCs initiate and co‐ordinate repair of a large destabilizing IVD lesion	162
Methodological review	Strategies for IVD repair repair using MSCs and bioscaffolds	135
IVDD induction by a postero‐lateral annular IVD lesion	Use of allogeneic mesenchymal precursor cells to promote healing in a postero‐lateral annular IVD lesion	205
Microdiscectomy IVDD model	MSCs plus PPS for regeneration of the degenerate IVD	206
Lumbar IVDD model	Immunoselected STRO‐3 + MSCs for IVD repair	207
Cervical model of IVDD	Use of MSCs + PPS to preserve cervical motion segments.	208

Other therapeutic procedures evaluated for repair of the ovine IVD and spinal defects		
Model	Features	References
In‐vitro and in‐vivo study	HA oligosaccharides stimulate MMP and anabolic gene expression in vitro and annular repair	163
Thoracic spine fusion model.	Development of an open mini‐thoracotomy surgical approach to an ovine thoracic spine fusion model.	209
Lumbar interbody spine fusion model	Augmented bone graft products demonstrated to compare favorably with autologous bone graft in an ovine model of lumbar interbody spine fusion	210
Anterior cervical discectomy and fusion models	Failure of resorbable plates and screws in an ovine model of anterior cervical discectomy and fusion.	211

## 12 ANIMAL MODELS DEVELOPED TO STUDY LOW BACK‐PAIN

Animal models have provided invaluable information of molecular events occurring during IVDD, and importantly have demonstrated how changes in the IVD microenvironment can lead to the development of discogenic LBP and pain in other spinal structures such as muscle, CEP, facet joint and spinal ligaments.^212^ Paraspinal muscles affected by IVDD are major pain centers in the spine.^212^ A rat multifidus transection model of IVDD shows the inter‐dependance of spinal muscles and the IVD.^213^ This is a useful model which utilizes an intact IVD and shows how induction of spinal instability can initiate IVDD without violating IVD structure directly. The altered biomechanical loading on the degenerate IVD and mechanical destabilization alters cell‐ECM signaling resulting in the production of inflammatory cytokines and active MMPs which further promotes disc degenerative process.^212^ Degenerative changes in the disc ECM conducive to neovascularization and the ingrowth of nerves and an elevation in the production of inflammatory cytokines and neurotrophic factors all lead to a significant deterioration in the normal cellular microenvironment in the IVD resulting in increased numbers of mechano‐and nociceptive pain receptors.^214^ Noxious stimuli in the IVD such as an acidic pH, ECM degeneration, inflammatory mediators and neurotrophins generate inflammatory conditions in the IVD that promote this increase in noci‐ and mechanoreceptors. These environmental changes in the degenerate IVD results in membrane depolarization of peripheral nociceptive nerve endings^215^, ^216^ and nerve activation and axonal transduction of such signals to somatic DRGs then to the dorsal horn sensory gray matter of the spinal cord and the brain. Signal transduction through chemical synapses in neural networks carries pain signals from IVD nociceptors to the brain.^217^ Activation of IVD neuronal activity by inflammatory mediators also induces protein kinase A and C, calcium/calmodulin‐dependent protein kinase, and MAPK signaling in dorsal horn neurons and the induction and maintenance of neuropathic pain. Activation of MAPKs (p38, ERK, and c‐Jun N‐terminal kinase) in spinal cord microglia or astrocytes results in the production of inflammatory mediators, sensitization of dorsal horn neurons and activation of spinal glia. Such neuron–glia interactions enhance and prolong neuropathic pain.^218^

LBP is a condition recognized as the leading musculoskeletal condition of major socioeconomic impact and a major cause of years lived with disability.^219^, ^220^, ^221^, ^222^ LBP can be ellicited by painful stimuli emanating from the spinal muscles, nerves, or spinal bones and is often exacerbated by IVDD.^223^ LBP can vary in intensity from a dull constant ache to a sudden sharp pain^224^ and is classified by its duration time as acute (pain duration <6 weeks), sub‐chronic (pain duration 6–12 weeks), or chronic (pain duration>12 weeks).^225^, ^226^, ^227^ LBP may be further classified by its underlying cause as mechanical, non‐mechanical, or referred pain.^228^ About 40% of the worlds human population suffer from LBP some time in their lifetime^229^ and this may be as high as a value frequently quoted for Western societies of 80%.^221^ It is estimated that 9–12% of the global general population (632 million) have LBP at any one time. LBP symptoms are often first evident between 20 and 40 years of age^223^ with men and women being equally affected and it is more common in individuals aged 40–80 years of age with the incidence of LBP increasing with advancing age.^229^, ^230^

Animal models continue to be developed for specific applications focussing on low LBP, mainly dominated by rodent studies however developments in the interpretation of pain responses in large animals has also emerged.^231^, ^232^, ^233^, ^234^, ^235^, ^236^, ^237^ A number of procedures have also been developed to assess pain responses in other large animal models of appendicular osteoarthritis.^232^, ^238^, ^239^, ^240^, ^241^ Significant advances in facial recognition technology with the application of AI and DML has also emerged as useful methodology in animal pain models, although it may be more suitable for acute rather than chronic painful conditions.^242^, ^243^, ^244^, ^245^, ^246^ The mouse continues to be a popular animal model for such IVD studies (see supplemental information) and a multitude of genetically modified mice have been developed to ask specific questions relevant to disc pathobiology (see supplemental information). A recent review of these mouse strains and their usefulness in studies on IVDD pathobiology demonstrates the invaluable contributions they have made to our understanding of molecular pathophysiology.^79^ Knowledge on the biology and potential usefulness in IVD regenerative procedures of notochordal cells gleaned from specific animal models of disc degeneration including mice has also increased significantly in the last 10 years.^132^, ^247^, ^248^, ^249^, ^250^, ^251^, ^252^

## 13 APPLICATION OF AI IN THE ANALYSIS OF IVDD, LBP AND PATIENT TREATMENTS

AI is a collection of digital technologies that have found widespread application in data analysis in many aspects of healthcare.^253^ Machine learning is a statistical technique that models data and has widespread application in precision medicine, predicting what treatments are likely to succeed on an individualized patient basis and can also be used in the assessment of outcomes from surgical procedures or the development of personalized rehabilitation protocols.^254^ DML or neural network analysis mimic the interactivity and cooperativity of the signaling evident in neural networks in the brain and are more complicated forms of machine learning. These have predictive capability and are amenable to automation increasing the throughput of spinal data analysis. From its inception in the late 1900 s AI has found application in a diverse range of areas in healthcare. Becker^255^ proposed that AI has proved useful in (i) risk assessments of disease onset and evaluation of the success of treatments, (ii) management or elimination of complications, (iii) patient oncare assessments, and (iv) research into the efficacy of treatments and the pathological evaluation of disease parameters. Merali and colleagues have used AI to predict outcomes from surgery to treat degenerative myelopathy.^256^ Karhade et al.^257^ developed machine learning algorithms to predict surgical outcomes of patients who received surgery to treat lumbar degenerative disc disorders. Deep learning techniques can predict the outcome of patients treated for lumbar disc herniation after a 6 month recovery period and can also be used to identify early stage patients likely to benefit from conservative therapy aiding in decision‐making for patient treatment options.^258^, ^259^

## 14 APPLICATION OF AI TO BETTER UNDERSTAND DEGENERATIVE PROCESSES IN PARKINSON'S DISEASE

AI has been applied in the assessment of Parkinson's disease (PD) in 1000 fibroblast cell lines from normal and PD affected individuals using robotic technology, automated cell culture and cell painting techniques to evaluate cellular structural changes in PD affected cells.^260^ AI has significantly improved evaluation of the complex molecular events occurring in PD.^260^ This is a good example of how AI may be potentially applied to aid in the resolution of a complex disease process and in the development of prospective therapeutic drugs. A similar approach could potentially be applied to better understand the complexities of IVDD and development of therapeutic drugs.

## 15 HOW AI AND DML HAVE IMPROVED CLINICAL SPINAL IMAGING

Clinical imaging of degenerative pathological features in disease processes yields invaluable information relevant to the diagnosis, management or development of therapeutic measures to treat such diseases.^261^ AI and DML are particularly well suited to the evaluation of large data sets in clinical imaging. A deep convolutional neural network trained on a large, manually evaluated data set of 1599 patients and 7948 IVDs has been developed and outperforms human evaluations of MRI data.^262^ A semantic segmentation network (BianqueNet) composed of three innovative modules achieves high‐precision segmentation of IVDD‐related regions.^263^ This quantitative method calculates the signal intensity and geometric features of IVDD strongly correlated with IVDD grade. This fully automated quantitation system provides precise clinical information for clinical trials, and investigations into the mechanism of IVDD, and the increased spinal imaging capability increases the throughput of patient data.^263^ A deep learning based program (Spine Explorer) has been developed for automated segmentation and quantification of the vertebrae, spinal muscles and IVDs in lumbar spine MRIs.^264^, ^265^, ^266^ Regions of vertebrae and discs are manually segmented on T2W sagittal MRIs used to train a convolutional neural network for automated segmentation and quantitative morphometric and signal measurements of lumbar vertebrae and discs. MRIs in this dataset can be automatically measured and manually checked with Image J.^264^, ^265^, ^266^ Integrating machine learning techniques have been developed to provide detailed and objective clinical assessments of IVDD effects on patient mobility.^267^ The five‐repetition sit‐to‐stand (5R‐STS) test is an objective tool for the testing of everyday flexibility and muscular co‐ordination in spinal movements and has found application in the evaluation of the validity and reliability of the 5R‐STS test in patients with degenerative pathologies of the lumbar spine and functional impairment induced by IVDD.^268^

Modic changes (MCs) are used to describe MRI signal intensity changes in vertebrae and correlated with disease pathology in degenerative spinal disorders. However, there is a growing need for novel quantitative and standardized methods to improve the interpretation of such Modic spinal changes in disease processes. A deep learning‐based approach to the analysis of Modic images has provided findings that are substantially in agreement with spinal evaluations by radiologists with the potential to improve inter‐rater reliability of such evaluations of MRI assessments.^269^ AI and DML are thus revolutionizing spinal imaging, including visualization of vertebrae and IVDs in diagnostic and surgical outcome and rehabilitation assessments, biomechanics, and motion analysis.^270^

DML has also improved the analysis of clinical spinal images in the last 6 years in the segmentation, pathology detection, diagnosis, and quantitative evaluation of MRIs.^271^ Positron emission tomography/computed tomography (PET/CT) of the lumbar spine using^18^ F‐fluoro‐deoxyglucose (FDG) and^18^ F‐sodium fluoride (NaF) tracers have imaged spinal inflammation and microcalcification, in degenerate spinal structures.^272^ Functional MRI and^18^ F‐FDG PET imaging have helped to define pain‐relevant, physiologically active brain regions.^273^ An increased uptake of^18^ F‐FDG in the caudal aspect of the LBP thoracic spinal cord locates metabolic activity in the spinal cord^273^, ^274^ This could aid in the treatment of LBP by localizing physiologically active cord regions to guide minimally invasive delivery of analgesics or stimulators to these regions. Changes in cerebral metabolic activity and multi‐frame static brain^18^ F FDG PET imaging after L2 DRG stimulation for discogenic LBP has also shown increased metabolic activity in nociceptive brain matrices and an increase in F^18^ F FDG uptake following DRG stimulation.^275^ Based on how DML has improved MRI of the spine its application to PET/CT imaging would also be expected to further improve on what is already a powerful imaging methodology.

## 16 APPLICATION OF AI IN ANIMAL MODELS OF IVDD

Mice are popular preclinical models to elucidate mechanisms of IVDD and the testing of potential therapeutics. Artificial intelligence and supervised and unsupervised machine learning algorithms, including artificial neural networks have been applied to quantitate degenerative changes identified using a new quantitative 14 category histopathological scoring scheme in murine IVDD, with high sensitivity and specificity.^276^ A 27 point 6 category histopathological scoring scheme has also been developed to quantitate specific degenerative features in an ovine experimental animal model of IVDD.^179^ This scheme demonstrated the efficacy of mesenchymal stem cells for the repair of AF lesions and regeneration of the IVD,^162^ and would be amenable for AI based evaluation similar to that used in the murine model. Implementation of AI methodology with the ovine IVDD model would improve its sensitivity and accuracy by obviating inter‐observer variation, and increase its utility for evaluation of novel therapeutic compounds. With ongoing development of more complex multi‐tissue pathology scoring schemes, evaluation of multiple pain outcome measures, generation of genome‐wide expressian data from numerous tissues, and use of new imaging technologies, application of AI and DML methods in animal models of IVDD will become increasingly important both to interrogate and interpret research findings and to translate these to equivalent human measures.

A recent publication employing AI has been applied to computational imaging of painful and pain free human IVDs.^277^ Using this approach in‐growth of blood vessels and nerves have been imaged and quantified in painful IVDs vindicating an earlier publication where it was demonstrated that an ingrowth of blood vessels and nerves occurred into degenerate IVDs when they became depleted of their space filling aggrecan proteoglycans.^37^ Furthermore this AI approach was used to develop a predictive tool and pain index for the assessment of macroscopic images of normal and degenerate human IVDs. These findings were confirmed using conventional confocal histology and histopathological scoring of the degeneracy grade of the tissues.^277^ An influx of inflammatory cells along fissures in the outer and inner AF associated with vessel ingrowth but only in painful IVDs was also demonstrated^277^ confirming earlier observations obtained using an ovine model of experimental disc degeneration.^37^, ^178^ This new AI methodology has thus improved on conventional imaging of the IVD.^277^

## 17 PROMISING NEW THERAPIES FOR IVDD AND LBP

Many compounds have shown considerable promise in countering the inflammatory conditions that occur in the degenerate IVD^278^ and which stimulate IVD repair processes^279^ in in‐vitro laboratory experiments and pre‐clinical studies (Table 4). These compounds warrant further evaluation in‐vivo and the aforementioned murine and ovine models would be suitable for this purpose and improved by the application of AI technology.

**TABLE 4:** Therapeutic compounds that show promise in the prevention of IVDD and LBP

Naturally occurring NP protective phytochemicals with an ability to inhibit IVDD		
Gefitinib	Gefitinib, a tyrosine kinase inhibitor, suppresses EGFR and has protective properties against IVDD in the rat IVD.	280
Atsttrin TNFα inhibitor of IVDD	Atsttrin, an engineered protein identified from studies on progranulin, inhibits TNF‐α‐induced inflammation and suppresses IVDD.	281
SIRT1, Sirtuin family member NAD‐dependent deacetylase	SIRT1 has protective effects in a puncture‐induced IVDD model. Resveratrol, a SIRT1 activator also protects against puncture‐induced IVDD.	282
Lycorine, alkaloid of lily family and daffodilsLycorine (*Lycoris radiate*) pyrrolophenanthridine	Suppresses MMP‐3, 13, ADAMTS‐4, 5 expression by CEP cells through prevention of NFκB signaling and IL‐1β‐induced endplate degeneration. Reduces proinflammatory cytokines, suppresses expression of MMP‐3, 13, ADAMTS‐4, 5 by CEP cells via inhibition of NFκB signaling.	283, 284
Tanshinone IIA	Reduces inflammation and radiculopathic pain, inhibits IRAK‐1 and NF‐κB/p38/JNK signaling and pro‐inflammatory mediator production, MMP expression, and radiculopathic pain.	285
Icariin	Attenuates IL‐1β‐induced inflammatory response in human NP cells.	286
Wogonin	Inhibits IVDD through upregulation of Nrf2/ARE and MAPK signaling.	287
Epigallocatechin 3‐gallate	Suppresses IL‐1β‐induced inflammatory responses in IVD cells in vitro and reduces radiculopathic pain in‐vivo.	288
Quercetin	Inhibits apoptosis of NP cells, alleviates IVDD by modulating p38 MAPK‐mediated SIRT‐1 autophagy pathway and the Nrf2/NF‐κB axis.	289, 290, 291
Diosmin, citrus flavonoid	Trialed for treatment of LBP, reduces neuropathic pain in mice/rat models.	292, 293, 294
Procyanidin B3 dimer	Aleviates IVDD via interaction with the TLR4/MD‐2 complex.	295
Luteolin	Inhibits IL‐1β‐induced NP apoptosis /catabolism, ameliorates IVDD.	296
Baicalein	Inhibits IL‐1β‐induced inflammation in NP cells in‐vitro and IVDD.	297
Hesperidin	Anti‐hyperalgesic properties in a rat neuropathic pain model, has anti‐oxidant and anti‐inflammatory properties.	298
Curcumol	Alleviates inflammation in NP cells via PI3K/Akt/NF‐κB signaling.	299
Mangiferin	Alleviates NP mitochondrial ROS, suppresses NF‐*κ*B signaling pathway.	300
Naringin, Naringenin	Protects human NP cells from TNF‐*α*‐induced inflammation, oxidative stress, enhanced AMPK/SIRT1 autophagy, IVD repair, reduces LBP.	301, 302, 303
Cannabidiol (CBD)	Alleviates pain via CB‐1, 2R, G protein serotonin R; TRPV‐1, PPAR, prevents anti‐oxidative stress, inflammation, senescent effects in NP. Suppresses MMP‐9, 13, induces Coll II, SOX‐9 via AMPK/GSK3β pathway.	304, 305, 306, 307, 308, 309, 310, 311, 312, 313
Celastrol pentacyclic quinone of *T. wilfordii*	Decreases MMP‐3, 9, 13, ADAMTS‐4, 5, COX‐2, iNOS, IL‐6 and TNF‐α in a rat IVDD model, inhibits the NF‐κB pathway in NP cells	314
Ligustilide (Ligustrazine) essential oil from *Ligusticum striatum*	Inhibits apoptosis, iNOS, COX‐2, TNF‐α/IL‐6 expression in NP cells, IVDD, suppresses aberrant TGF*β* activation in NP cells, anti‐inflammatory, reduces IVDD and neurogenic pain.	314, 315
Ganoderic acid A, hetero‐ cyclic triterpenoid of *Ganoderma lucidum* fungi	Suppresses activation of TLR4/NLRP3 signaling, inhibits H_2_O_2_ induced apoptosis, inflammatory cytokines and oxidative stress, up‐regulates Col II and aggrecan production by NP cells.	316
Animal metabolites with pain relieving properties		
Resolvin D1 (RvD1)	RvD1 is a pro‐resolving lipid mediator that reduces neuropathic pain. SC stimulation reduces CSF IL‐1β levels and increases RvD1 levels alleviating IL‐1β induced neuropathic pain and neuroinflammation.	317, 318
Hemorphins	Hemorphin LVV‐H7 has analgesic effects in a model of SC injury, through IL‐1 blockade by IL‐R antagonist protein and activation of opioid receptors.	319

Abbreviations: Akt, serine/threonine‐specific protein kinase; AMPK, 5′ AMP‐activated *protein* kinase; CSF, cerebrospinal fluid; EGFR, epidermal growth factor receptor; GSK3β, Glycogen synthase kinase‐3 beta; IRAK‐1, interleukin‐1 receptor‐associated kinase‐1; JNK, c‐Jun N‐terminal kinase; NFκB, Nuclear factor kappa B; Nrf2, Nuclear factor erythroid 2‐related factor 2/ARE and MAPK, mitogen‐activated protein kinase; MD‐2, lymphocyte antigen 96; PI3K, phosphatidyl‐3 kinase; PPAR, peroxisome proliferator‐activated receptor; ROS, reactive oxygen species; SIRT‐1, NAD‐dependent deacetylase *sirtuin*‐*1*; SC, spinal cord; TLR4, Toll‐like receptor‐4; TRPV‐1, transient receptor potential cation channel subfamily V member 1.

## 18 ANTI‐INFLAMMATORY PHYTOCHEMICALS DISPLAYING IVD CELL PROTECTIVE IVD REGENERATIVE PROPERTIES AND THAT HAVE PAIN RESOLVING CAPABILITY APPLICABLE TO THE TREATMENT OF LBP

Flavonoids are a widely distributed family of polyphenolic dietary plant compounds that possess anti‐oxidant and anti‐inflammatory properties through their abilities to inhibit LOX, COX, NFκB, and iNOS activity [reviewed in ^320^]. They also induce nuclear factor‐erythroid factor 2‐related factor 2 (Nrf2) gene expression in cells. Nrf2 is a master transcription factor that regulates the expression of over 1000 genes in the cell under normal and stressed conditions. Nrf2 induces expression of an array of antioxidant response element‐dependent genes that regulate physiological and pathophysiological outcomes of oxidant exposure providing cell and tissue protective properties during damaging oxidizing cellular environments. Flavonoids may well turn out to be useful neutraceutical supplements for IVD protection but further research is required to properly assess this possibility. A selection of flavonoids and related phytochemicals with anti‐oxidant and anti‐inflammatory properties and in some cases an ability to promote IVD regeneration and alleviate pain are presented in Table 4. The recent identification of the gut microbiome‐IVD axis may be a potential delivery system for potent anti‐oxidant and anti‐inflammatory gut generated phytochemical metabolites with cell and tissue protective properties^321^, ^322^, ^323^ and potential roles in the prevention of chronic pain.^324^, ^325^, ^326^, ^327^ Further studies are warranted to examine this possibility.

Flavonoids have anti‐oxidant and anti‐inflammatory properties that modulate the production and action of inflammatory cytokines and provide benefit in the treatment of OA and RA.^328^, ^329^, ^330^, ^331^ Several flavonoids have also shown promise in the treatment of IVDD^287^, ^291^, ^297^, ^301^ and in pain alleviation.^74^, ^332^, ^333^, ^334^, ^335^, ^336^, ^337^, ^338^ Wogonin mitigates IVDD through the Nrf2/ARE and MAPK signaling pathways.^287^ Quercetin alleviates IVDD by modulating p38 MAPK‐mediated autophagy.^291^ Baicalein inhibits the IL‐1β‐induced inflammatory response in NP cells and attenuates disc degeneration in vivo.^297^ Lycorine is a major alkaloid pyrrolophenanthridine component of the traditional medicinal Chinese herb amaryllidaceae family *Lycoris radiate*. Lycorine displays strong anti‐leukemia, anti‐tumor, anti‐angiogenic, anti‐viral, anti‐bacterial, anti‐inflammatory, and antimalarial pharmacological properties.^321^ Lycorine suppresses the expression of MMP‐3, 13, ADAMTS‐4, 5 by cells of the CEP via the inhibition of NFκB signaling preventing IL‐1β‐induced CEP and NP degeneration in‐vitro.^283^ Lycorine reduces proinflammatory cytokine production and protects cartilage from degradation by MMPs in a mouse model of OA.^339^, ^340^ Diosmin is a citrus flavonoid (diosmetin 7‐O‐rutinoside) non‐prescription dietary supplement that has been trialed for the treatment of LBP.^294^ Diosmin is reported to reduce chronic constriction injury‐induced neuropathic pain in mice^292^ and provides central and peripheral anti‐hyperalgesic effects in a neuropathic pain model in rats.^293^ The related flavonoid hesperidin has anti‐hyperalgesic properties in a neuropathic pain model in rats.^298^ Further studies on flavonoids are warranted to determine their optimal mode of administration, mechanism of action, specific molecular targets and how efficacious drug levels in specific tissues can best be obtained. Tanshinone IIA represses inflammatory responses and reduces radiculopathic pain by inhibiting IRAK‐1 and NF‐κB/p38/JNK signaling.^285^ Tanshinone IIA displays potent anti‐inflammatory and anti‐catabolic activities, and is an abundant component of the root of the Chinese sage (*Salvia miltiorrhiza*) highly prized in traditional medicine. Tanshinone IIA inhibits the expression of pro‐inflammatory mediators and MMPs in‐vitro, and radiculopathic pain in‐vivo, and displays potential in the treatment of inflammation and the generation of LBP In IVDD. Quercetin inhibits senescence associated secreted phenotypic factor expression in NP cells and ameliorates IVDD via the Nrf2/NF‐κB axis,^289^ suppresses NP cell apoptosis and attenuates IVDD via the SIRT1‐Autophagy Pathway.^290^ Procyanidin B3 (Pro‐B3), a catechin dimer biflavonoid is a common component of the human diet which has anti‐inflammatory properties, inhibiting LPS‐mediated ECM degradation and the activation of the NF‐κB/TLR‐4 pathway in NP cells.^295^ Naringin and naringenin are flavonoids that regulate cytokine, MMP, ECM protein gene expression and genes that promote apoptosis of NP cells. Molecular docking studies have demonstrated binding of naringin and naringenin to genes identified as potent inhibitors of inflammation. Collectively these traits show the beneficial properties of naringin and naringenin in ECM and NP cell protection in IVDD and LBP.^302^, ^303^

Mitochondrial dysfunction promotes IVDD by affecting oxidative stress, mitophagy, mitochondrial homeostasis, cellular senescence, cell death and metabolic dysfunction.^341^ Flavonoids modulate antioxidant cellular responses, apoptosis, mitochondrial biogenesis, autophagy, and have roles in mitochondrial ion channels that can be cytoprotective.^342^, ^343^ Flavonoids have beneficial effects on mitochondrial homeostasis through their inherent anti‐oxidant properties.^342^ The cell protective properties of flavonoids is partly due to their ability to counter mitochondrial mysfunction.^320^, ^344^ Urolithin A is a benzo‐coumarin metabolite produced by the gut microbiome by digestion of ellagic acid and ellagitannins found in dietary pomegranates, strawberries, raspberries and wallnuts. Urolithin A does not occur free in dietary foods and is not produced by mammalian enzyme systems.^345^, ^346^ Urolithin A is a natural pro‐biotic that promotes mitophagy, mitochondrial biogenesis and metabolic function impacting on muscle health in preclinical models of aging and in the elderly and middle‐aged. Urolithin A improves mitochondrial function in the articular chondrocytes of diarthrodial joints, reduces disease progression in a mouse OA model and inhibits cartilage degeneration, synovial inflammation, and the pain associated with this condition.^347^ Urolithin A thus has properties that would also be beneficial in the treatment of IVDD and LBP. The gut microbiome‐IVD axis proposed by Li et al.^348^ and Rajasekaran et al.^349^ may serve as an intrinsic delivery system that could be utilized to deliver efficacious gut metabolites such as urolithin A to the IVD in a similar manner to how bioactive gut metabolites are transported by the gut brain axis.^350^, ^351^, ^352^ This also gives some credibility to the consumption of diets enriched in probiotic compounds as a potential therapeutic option to promote tissue repair. This proposal warrants further investigation.

Cannabidiol (CBD) displays potential in the management of neuropathic LBP. CBD has antidepressant, anxiolytic, anticonvulsant, antipsychotic and pain alleviating properties. CBD acts through the cannabinoid CB1 and CB‐2 receptors, three G protein coupled‐receptors (adenosine receptor subtype 2A, serotonin receptor subtype 1A and G protein‐coupled receptor 55), one ligand‐gated ion channel (transient receptor potential vanilloid channel‐1, TRPV‐1) and one nuclear factor (peroxisome proliferator‐activated receptor γ, PPAR). CBD has been used in the management of epilepsy,^353^, ^354^ Alzheimer's disease,^355^ pain alleviation in MS,^307^ neurological responses in Parkinson's disease,^308^, ^313^ chronic pain^305^, ^306^, ^309^, ^312^ including LBP.^311^ CBD has been evaluated in a double blind randomized clinical trial for the relief of central neuropathic pain in MS.^307^

## 19 MAMMALIAN AGENTS WITH PAIN ALLEVIATING PROPERTIES

Progranulin has roles in the regeneration of peripheral nerves following trauma^356^, ^357^ its overexpression in sensory neurons attenuates neuropathic pain in mice^356^ through effects on autophagy. Increased autophagic activity in dorsal root ganglia also attenuates neuropathic pain following peripheral nerve injury.^358^ Animal models of inflammatory and neuropathic pain show inflammation regulates pain resolution by producing pro‐resolving mediators such as resolvin D1 (RvD1).^318^ Resolvins are derived from, eicosapentaenonic, docosahexanoic, docosapentaenoic, and clupanodonic omega‐3 fatty acids^317^, ^359^, ^360^ and have cell regulatory properties similar to prostaglandins promoting the restoration of normal cellular functional properties following resolution of inflammatory conditions that occur with tissue injury. Resolvins are induced in the CNS following trauma and offer exciting possibilities in the development of potential methods for the alleviation of intractable neuropathic pain in chronically affected patients.^317^, ^359^, ^360^ The inter‐relationship between inflammation and neuropathic pain needs to be resolved to provide pain relief in musculoskeletal disorders.^361^ Resolvins are lipid mediators that are released during the resolution phase of acute inflammation. The resolvins are active at pico to nanogram levels and have the ability to regulate proinflammatory processes actively promoting monocyte and macrophage uptake of cellular and ECM debris, apoptotic PMNs, and the killing and clearing of invading microbes.^362^ The resolvins also reduce the activation of CD4 and CD8 cell activation and prevent Th1 and Th17 cell differentiation^363^ and have potent anti‐inflammatory and pain resolving properties.^364^ Aspirin can initiate resolvin production as part of its pain resolving properties.^365^ The antiinflammatory and pro‐resolution properties of RvD1 offers novel therapeutic possibilities in the management of neuropathic pain.^366^ Resolvins alleviate neuropathic pain by regulating inflammatory mediators involved in the NF‐κB/p65 and p‐ERK cell signaling pathways and inhibit TRPA1, TRPV3 and TRPV4 activity correlating with changes in acute pain behavior in animal models consistent with pain relief.^367^ Resolvin D1 and fish oil n‐3 polyunsaturated fatty acids stimulate neurite outgrowth from primary DRG neuron cultures in normal mice. Dietary supplementation with fish oil or daily RvD1 in a mouse model of type 2 diabetes significantly improved diabetic neuropathy.^368^ Electrostimulation of the spinal cord induces RvD1 production, lowers production of inflammatory mediators and contributes to pain relief.^318^

In the last three decades, a number of studies on bioactive peptides that are opioid receptor ligands, have been undertaken.^369^ Hemorphins are endogenous 4–10 amino acid peptides released during proteolysis of the beta subunit of hemoglobin. The hemorphins exhibit diverse therapeutic effects in both humans and animal models including regulation of blood pressure, mood regulation, enhancement in memory, and cognitive learning and analgesic effects.^370^, ^371^, ^372^ Such effects occur through the ability of these peptides to modulate a diverse range of proteins including enzymes and G‐protein coupled opioid receptors.^371^ The resolvins and hemomorphins offer considerable promise as potential agents that can be developed for the alleviation of chronic neuropathic and nociceptive LBP in the future.

## 20 NOTOCHORDAL CELL‐BASED THERAPEUTICS FOR THE TREATMENT OF IVDD

### 20.1 The notochord and IVD development

Vertebrates are classified within the phylum Chordata, and are named because during later embryonic development, the notochord is replaced by the vertebral column with, depending on species, remnants of the notochord persisting within the IVD nucleus pulposus (NP) for variable periods.^373^ Lineage tracing studies have demonstrated that all cells within the NP originate from the notochord.^374^ With maturity in many mammals including humans, the cells within the IVD NP become chondrocyte‐like and lose their notochordal phenotype, presumably due to differentiation.^375^, ^376^ During development a multitude of signals from the notochord influence the fate of undifferentiated mesenchymal tissues (paraxial mesoderm), particularly the spinal cord that is formed as a consequence of condensation of neural crest cells that reside dorsal to the notochord that form the neural tube and ultimately the spinal cord.^377^, ^378^ Central to dorsal embryonic cellular and tissue fate, sonic hedgehog (SHH) concentration‐dependent differentiation of dorsal structures figures prominently however there are other mitogens and morphogens at play such as BMP2 and BMP4.^379^ In support of these vital developmental decisions on the part of undifferentiated cells/tissue, grafting of ectopic notochordal tissues dorsal to the neural tube leads to inhibition of normal dorsal structure tissue formation.^379^ Nonetheless and importantly with respect to the IVD, the terminal stage of vertebrogenesis in higher animals is marked by segmentation of the notochord where notochordal remnants persist within the center of the NP.^380^

The brachyury gene “T” codes for the T‐box transcription factor that along with *SHH*, *Noggin* and Paired box (*Pax*)*1* is involved with sclerotomal differentiation and is necessary for notochord maintenance.^381^, ^382^ SRY‐related HMG‐box (Sox) transcription factors, notably *Sox5*, *6* and *9* significantly influence the notochord post sclerotome formation with their expression similar to that occuring with chondrogenesis.^383^ Amongst the host of differentiation‐inducing factors, *Sox5* and *6* are considered to be crucial to the survival of notochordal cells within the NP such that homozygous *Sox5*−/− and *6*−/− mice develop abnormal, notochordal cell‐poor NPs with deformed vertebral columns.^383^ The precise molecular mechanisms involved with the ultimate destination of notochordal cells within the NP remain unknown, however much has been determined with respect to the function of these cells.

### 20.2 Importance of notochordal cells to IVD health

Interestingly, pigs, rabbits and non‐ChD dogs retain their notochordal cell‐rich NP throughout life and concomitantly do not normally develop IVDD. On the other hand, ChD dogs (beagles, Shih Tzus, Welsh Corgis, French Bulldogs and others) like humans, lose their notochordal cells in early life and are known to suffer early and significant IVDD. A recent paper has reported that ChD dogs have a CDDY mutation involving a second fibroblast growth factor (FGF)‐4 retrogene insertion in chromosome 12 that results in their short leg, longer torso phenotype.^384^ The different ChD and non‐ChD canine subspecies provides an opportunity to determine the relative contribution of the notochordal cell‐rich NP to protection from IVDD as compared with one that loses most if not all notochordal cells. Since the original paper by Aguiar et al. in 1999, a number of publications have emerged suggesting that notochordal‐cell secreted factors may be at least partially responsible for the protection from IVDD in non‐ChD canines.^125^, ^126^, ^248^, ^385^, ^386^, ^387^, ^388^, ^389^ Recently the secretome of the non‐ChD canine notochordal cell‐rich NP was analyzed.^126^ This study identified Connective Tissue Growth Factor‐(CTGF) and Transforming Growth Factor Beta‐1 (TGF‐β1) as key molecules capable of reducing the progression of IVDD in a small animal needle‐puncture induced model of IVDD. Most recently a single injection of these same molecules (suspended within an excipient solution), was shown to modify the progression of IVDD in small, and large animal models in vivo, and induce a regenerative effect in human IVD NP cells in vitro.^132^

It is interesting to note that it has been recently reported that injection of notochordal cell‐derived matrix (obtained from porcine IVD NPs rich in notochordal cells) into moderately degenerative ChD canines had a protective effect. In this report although the details of the injected notochordal cell‐derived matrix are lacking, the in vitro work utilized NP tissue that was lyophilized overnight, pulverized and then suspended within growth media and supplemented with antibiotics, L‐proline, ITS, ascorbic acid and bovine serum albumin.^390^ The essential element from this report is that capturing notochordal cell‐secreted products contributes to NP homeostatic regulation. Interestingly, the notion of understanding notochordal cell biology with respect to potential influence upon chondrocyte‐like cells within the NP to improve extra‐cellular matrix (ECM) integrity was postulated two decades ago by Oegema.^390^ Other animals are known to retain notochordal cells within their NPs such as rats, rabbits, cats and pigs and these animals also do not suffer from early IVDD, an observation that furthers the association of the notochordal cell‐rich NP and resistance to degeneration.^1^, ^140^, ^391^

Notochordal cells reside within the IVD NP as tightly packed physaliferous cells within a well hydrated, mucoid ECM rich in proteoglycans.^29^, ^125^, ^126^, ^132^, ^385^, ^386^, ^387^, ^388^, ^389^, ^392^ On the other hand, the cells within the ChD canine IVD exist as clusters of chondrocyte‐like cells within a more fibrocartilaginous ECM. Non‐ChD and ChD canines produce and assemble proteoglycans differently.^392^ Notochordal‐cell‐secreted glycoproteins in non‐ChD IVDs rapidly migrate to the inter‐cellular compartment prior to forming large sized aggregates. On the other hand, PGs synthesized by chondrocyte‐like cells in the ChD IVD are synthesized at a lower rate and form large aggregates within the pericellular region and only slowly moving to the inter‐cellular space over many days thereafter. One important conclusion from this work of Cappello et al. is that the cellular phenotype of the IVD NP has significant impact upon tissue composition and integrity.^392^

### 20.3 Clinical translation of notochordal based therapeutics

The burden of IVDD and associated spinal pain is significant with chronic back pain causing the highest years lost to disability globally of any condition.^221^ While IVDD is present in asymptomatic patients, large population studies involving patients suffering from back pain demonstrate significant positive correlation between their back pain and severity of IVDD on MRI.^393^ A recent report indicated a single injection of rhTGF‐β1 plus rhCTGF/CCN‐2 within an excipient conditioned medium offered good pre‐clinical evidence that this combination of molecules (identified from the notochordal‐rich IVD NP secretome) may be effective in modifying the course of IVDD.^125^, ^126^ Appropriate toxicological and phase 1 and 2 studies must be performed to further evaluate the efficacy and safety of this intervention in humans.

### 20.4 Translational challenges

In order to treat the degenerative human IVD, the most likely route of administration of any biologic would be a trans‐annular injection directly into the NP. Central to the notion of intradiscal injection is an accurate diagnosis of the symptomatic disc since most cases of IVDD have adjacent sites showing degenerative change that may or may not also be symptomatic. An emerging technology that may mitigate potential risks associated with discography and needle puncture concerns the use of magnetic resonance spectroscopy (MRS). This technology is reported to have an accuracy of 85%, sensitivity of 82%, and specificity of 88% with respect to identifying the painful disc as compared with provocative discography.^394^ Although still investigative, MRS may prove to be a preferred method (along with other clinical, laboratory and imaging methods) to noninvasively diagnose the symptomatic disc and aid in therapeutic decision making. Biologic therapy for the painful IVD would revolutionize the treatment of this disabling and expensive condition.

## 21 CONCLUSIONS

While there is no question that animal models have provided insightful information on the pathobiology of IVDD and the identification of therapeutic molecular targets applicable to the human condition, it is clear that this is a very complex and multifactorial disease process. It is also clear that animal models in isolation are incapable of providing an answer to the many complex features of human IVDD as a structural disease and an illness with variable pain types and states, and functional disability. AI can deal with complex large data sets, optimal results are obtained from big data where outcome measures can be clearly set. AI may represent the next step forward in the development of an IVDD algorithm which makes sense of IVDD, a very complex disease. AI and DML have already improved the analysis of clinical spinal imaging used to diagnose and assess the development of IVDD. AI may lead to the development of effective therapeutic measures for IVDD and a means of evaluating the success or failure of any prospective treatment procedure for a major healthcare problem in modern day society. AI has also been used in the development of screening procedures for the many potential therapeutic compounds listed in Table 4 and in the identification of their molecular targets. AI is also being used in the development of novel therapeutic drugs of high efficacy. The identification of the utility of murine and ovine IVD models for therapeutic drug evaluations, may thus find application using AI and DML in LBP and IVDD research. There is considerable promise that AI methodology could find application in unraveling the considerable complexities of IVDD and how best to treat this debilitating condition.

While currently there is a disconnect to some extent between AI and the novel therapies covered in this review the sheer depth of these studies outlining the beneficial tissue and cell protective properties of the many listed compounds and the illustration of how they might potentially be applied to IVD regeneration is highly suggestive of the likelihood that they may find future application. For this to become a reality more reliable evaluative methods are required for the assessment of IVD tissues and aspects of their regeneration in animal models. We consider that the AI methodology we have described in this review will provide such an improvement that may well allow the therapeutic potential of these novel compounds to be realized in future studies.

## AUTHOR CONTRIBUTIONS

This study was conceptualized by Mauro Alini and James Melrose with intellectual input from Chirstopher B. Little, Ashish D. Diwan, and W. Mark Erwin. All authors contributed to manuscript writing and review and all approved the final version of the manuscript.

## FUNDING INFORMATION

James Melrose has received consultancy fees from Arthropharm and Sylvan Pharmaceutical companies. ADD's institutions receive unrestricted research grants from Nuvasive Australia & Baxter Inc., Education support from Globus Medical (PA). ADD receives payments from Cartago Biotech, educational consultant payments from 3M & Nuvasive, research service payments from Kunovus Technologies & Merunova. These companies had no impact or say in the design or content of this study.

## CONFLICT OF INTEREST

The authors declare no conflict interest.

## Supporting information


**APPENDIX S1:** Supporting Information.
