# Supplementary material for: An update on animal models of intervertebral disc degeneration and low back pain: Exploring the potential of artificial intelligence to improve research analysis and development of prospective therapeutics
Source: JOR Spine. 2023 Jan 30;6(1):e1230. doi: 10.1002/jsp2.1230 (PMC10041392; doi:10.1002/jsp2.1230)
Supplement: Supplementary file 1 — APPENDIX S1: Supporting Information. [file JSP2-6-e1230-s001.docx]

**Supplementary material.**

**Completion of canine genome**

The ‘dog’ genome was first sequenced in 2005 based on a purebred female boxer and was published in *Nature Communications*, this dataset is freely available online through the [National Center for Biotechnology Information](https://www.ncbi.nlm.nih.gov/bioproject/PRJNA448733) and formed part of a much larger study, *the*[*Dog10K*](http://www.dog10kgenomes.org/)*sequencing project*[^398^](#_ENREF_398), an international collaboration initiated to amass 10,000 canine whole genome sequences for reference and analysis.The whole genome sequence was completed in 2015 based on analysis of 722 canines sampled from 144 modern breeds, plus 54 wild canids and 100 village dogs [^399^](#_ENREF_399)^,^[^400^](#_ENREF_400).

Canine genomic sequence data can be viewed through GenBank ([**www.ncbi.nih.gov/Genbank**](http://www.ncbi.nlm.nih.gov/Genbank)) at NIH's National Center for Biotechnology Information (NCBI); EMBL Bank ([**www/ebi.ac.uk/index.html**](http://www.ebi.ac.uk/embl/index.html)) at the European Molecular Biology Laboratory's Nucleotide Sequence Database; and the DNA Data Bank of Japan ([**www.ddbj.nih.ac.jp**](http://www.ddbj.nig.ac.jp/)); UCSC Genome Browser ([**www.genome.ucsc.edu**](http://www.genome.ucsc.edu/)) at the University of California at Santa Cruz and the Ensembl Genome Browser ([**www.ensembl.org**](http://useast.ensembl.org/index.html)) at the Wellcome Trust Sanger Institute in Cambridge, England.

**The Ancestral *Ovis orientalis* breed and establishment of modern Merino sheep breeds.**

Sheep domestication from its wild ancestor, the mouflon (*Ovis orientalis*) began in SW Asia more than 11000 years BC [^401^](#_ENREF_401). These sheep reached Europe and the Mediterranean regions ca. 6000 BC [^402^](#_ENREF_402). In the 18^th^ century, purebred Merino rams were exported to Saxony (Germany) and France establishing the Merinolandschaf and Rambouillet breeds. Rambouillet rams were subsequently exported to Central Italy and gave rise to the Sopravissana breed [^403^](#_ENREF_403). Dispersal of Merino sheep to Eastern Europe began in the 18^th^ century. Merino sheep breeding began in Hungary in 1774 from 300 Merinos imported from Spain [^404^](#_ENREF_404) In China, fine-wool sheep breeds were developed from Mongolian/Tibetan ewe crosses with Soviet Merino and Rambouillet rams [^405^](#_ENREF_405). In 1797 Spanish pedigreeMerinos were exported to Australia and New Zealand by General Macarthur and the First Fleet, prior to that fat tailed sheep were the most common sheep breed in Australia. Composite fine-wool sheep breeds were established in the Soviet Union from Australian Merino, American Rambouillet, Merinolandschaf crossed with local Merino breeds (Novocaucasian and Mazaev Merino) [^406^](#_ENREF_406). Historically, the Merino and derived breeds that established the Australian fine-wool sheep flock have been important to the Australian and New Zealand economies and a lot of effort has been made to maintain and improve sheep genetics in Australia. The Macarthur merino originally exported to Australia from Spain was a relatively small framed sheep approximately half the size of the modern day merino which has been bred for improved wool production. The Australian flock continues to be improved to the present day with sheep bred with improved high micron fibre wool quality. In contrast, in the last 20-30 years, European Merino’s have experienced a dramatic decline in numbers to the point where they are now considered an endangered breed. Ciani et al and the sheep genomics consortium (2015) have examined the genetic diversity, structure and genetic traits of Merino and Merino-derived breeds on an inter-continental basis [^407^](#_ENREF_407).

**Mouse genome**

The laboratory mouse is the premier animal for investigations on IVD molecular and cellular systems [^408^](#_ENREF_408)^,^[^409^](#_ENREF_409). Many experimental genetic tools have been developed for the mouse, including unique inbred strains, a complete reference genome, deep sequencing data for 17 additional inbred lines [^410^](#_ENREF_410), extensive genome variation maps (e.g. SNPs), and technologies for genome manipulation [^411^](#_ENREF_411)^,^[^412^](#_ENREF_412). An international collaborative effort to generate targeted mutations in all murine protein-coding genes was initiated in 2007 [^413^](#_ENREF_413) and a draft genome has now been completed [^414^](#_ENREF_414), the phenotyping phase of functional characterization of these genes is now being undertaken [^415^](#_ENREF_415).The Mouse Genome Database (MGD; http://www.informatics.jax.org) is the primary community database for the laboratory mouse and a key source of gene biological reference data, gene functions, phenotypes, disease models relevant to human biology and disease freely accessible to all researchers. The short generational times, ease of genetic manipulation and relatively low cost of maintenance of large mouse colonies makes the mouse the animal model of choice for genetic studies.

**Standardised guidelines for animal model intervertebral disc studies**

In an effort to standardize experimental procedures using intervertebral disc animal models to provide more accurate valid comparisons between laboratories a series of studies have recently been published providing guidelines on the histopathological scoring of human [^416^](#_ENREF_416), rabbit [^417^](#_ENREF_417), rat [^418^](#_ENREF_418) and mouse [^278^](#_ENREF_278) intervertebral discs. The present review advocates merino sheep (*ovis aries*) as an appropriate large animal breed for intervertebral disc studies aimed at investigating controlled experimental intervertebral disc degeneration and therapeutic procedures for its repair. Studies have been published on how to create a controlled standardized annular lesion to induce intervertebral disc degeneration in sheep [^163^](#_ENREF_163). This study also outlines the degenerative features that are obtained using this procedure. A further study outlines the development of a quantitative histopathological scoring scheme for the sheep which can be used to evaluate degenerative as well as regenerative features using the sheep model [^189^](#_ENREF_189). The utility of bone marrow derived mesenchymal stromal stem cells for the repair of controlled experimental annular lesions has also been demonstrated in a further study where the degenerative features of this ovine model and regenerative features induced by stem cell treatment were quantitatively scored using this histopathological scoring scheme [^164^](#_ENREF_164).

REFERENCES

398 Wang, G., Larson, G, Kidd, JM, vonHoldt, BM, Ostrander, EA, Zhang, YP. . Dog10K: the International Consortium of Canine Genome Sequencing. . *Natl Sci Rev***6**, 611-613 (2019).

399 Lindblad-Toh, K., Wade, CM, Mikkelsen, TS, Karlsson, EK, Jaffe, DB, Kamal ,M, Clamp, M, Chang, JL, Kulbokas, EJ 3rd, Zody, MC, Mauceli, E, Xie, X, Breen, M, Wayne, RK, Ostrander, EA, Ponting, CP, Galibert, F, Smith, DR, DeJong, PJ, Kirkness, E, Alvarez ,P, Biagi, T, Brockman, W, Butler, J, Chin, CW, Cook, A, Cuff ,J, Daly, MJ, DeCaprio, D, Gnerre, S, Grabherr ,M, Kellis, M, Kleber, M, Bardeleben, C, Goodstadt, L, Heger, A, Hitte, C, Kim, L, Koepfli,KP, Parker, HG, Pollinger, JP, Searle, SM, Sutter, NB, Thomas, R, Webber ,C, Baldwin, J, Abebe, A, Abouelleil, A, Aftuck, L, Ait-Zahra, M, Aldredge, T, Allen, N, An, P, Anderson, S, Antoine, C, Arachchi, H, Aslam, A, Ayotte, L, Bachantsang, P, Barry, A, Bayul, T, Benamara, M, Berlin, A, Bessette, D, Blitshteyn, B, Bloom, T, Blye, J, Boguslavskiy, L, Bonnet ,C, Boukhgalter, B, Brown, A, Cahill, P, Calixte, N, Camarata, J, Cheshatsang, Y, Chu, J, Citroen, M, Collymore, A, Cooke, P, Dawoe, T, Daza, R, Decktor, K, DeGray, S, Dhargay, N, Dooley, K, Dooley, K, Dorje, P, Dorjee, K, Dorris, L, Duffey N, Dupes, A, Egbiremolen, O, Elong, R, Falk, J, Farina, A, Faro, S, Ferguson, D, Ferreira, P, Fisher, S, FitzGerald, M, Foley, K, Foley, C, Franke, A, Friedrich, D, Gage, D, Garber, M, Gearin, G, Giannoukos, G, Goode, T, Goyette, A, Graham, J, Grandbois, E, Gyaltsen, K, Hafez, N, Hagopian, D, Hagos, B, Hall, J, Healy, C, Hegarty, R, Honan T, Horn, A, Houde, N, Hughes, L, Hunnicutt, L, Husby, M, Jester, B, Jones, C, Kamat A, Kanga B, Kells C, Khazanovich D, Kieu AC, Kisner P, Kumar M, Lance, K, Landers, T, Lara, M, Lee, W, Leger, JP, Lennon, N, Leuper ,L, LeVine, S, Liu, J, Liu, X, Lokyitsang, Y, Lokyitsang, T, Lui, A, Macdonald, J, Major, J, Marabella, R, Maru, K, Matthews, C, McDonough, S, Mehta, T, Meldrim, J, Melnikov, A, Meneus, L, Mihalev, A, Mihova, T, Miller, K, Mittelman, R, Mlenga, V, Mulrain, L, Munson, G, Navidi, A, Naylor, J, Nguyen, T, Nguyen, N, Nguyen, C, Nguyen, T, Nicol, R, Norbu, N, Norbu, C, Novod, N, Nyima, T, Olandt, P, O'Neill, B, O'Neill, K, Osman, S, Oyono, L, Patti, C, Perrin, D, Phunkhang, P, Pierre, F, Priest, M, Rachupka, A, Raghuraman, S, Rameau, R, Ray, V, Raymond, C, Rege, F, Rise, C, Rogers, J, Rogov, P, Sahalie, J, Settipalli, S, Sharpe, T, Shea, T, Sheehan, M, Sherpa, N, Shi, J, Shih, D, Sloan J, Smith, C, Sparrow, T, Stalker J, Stange-Thomann, N, Stavropoulos, S, Stone, C, Stone, S, Sykes, S, Tchuinga, P, Tenzing, P, Tesfaye, S, Thoulutsang, D, Thoulutsang, Y, Topham, K, Topping, I, Tsamla, T, Vassiliev, H, Venkataraman, V, Vo, A, Wangchuk, T, Wangdi, T, Weiand, M, Wilkinson, J, Wilson A, Yadav, S, Yang, S, Yang, X, Young, G, Yu, Q, Zainoun, J, Zembek, L, Zimmer, A, Lander, ES. . Genome sequence, comparative analysis and haplotype structure of the domestic dog. . *Nature***438**, 803-819 (2005).

400 Neff, E. P. Whole genome sequencing has gone to the dogs. *Lab Anim* **48**, 166 (2019).

401 Demirci, S., Baştanlar, EK, Dağtaş, ND, Pişkin, E, Engin, A, Özer, F, Yüncü, E, Doğan, SA, Togan, I. Mitochondrial DNA diversity of modern, ancient and wild sheep (Ovis gmelinii anatolica) from Turkey: New insights on the evolutionary history of sheep. *PLoS ONE***8**, e81952 (2013).

402 Pereira, F., Davis, SJ, Pereira, L, McEvoy, B, Bradley, DG, Amorim, A. Genetic signatures of a Mediterranean influence in Iberian Peninsula sheep husbandry. *Mol Biol Evol***23**, 1420-1426 (2006).

403 Lasagna, E., Bianchi,M, Ceccobelli, S, Landi, V, Martinez, A, Pla, JLV, et al. Genetic relationships and population structure in three Italian Merino-derived sheep breeds. *Small Ruminant Res***96**, 111-119 (2011).

404 Fésüs, L., Sáfár L, Hajduk P, Székely P. in *Proceedings of the 6th Merino World Conference: 29 April – 1 May 2002.*

405 Philippine Council for Agriculture, F. a. N. R., Research and Development; International Development Research Centre. (ed C. Devendra and P.S. Faylon;).

406 Semyonov, S., Selkin, II. *Sheep: Animal genetic resources of the USSR.*, 154-271 (FAO-UNEP, 1989).

407 Ciani, E., Lasagna, E, D'Andrea, M, Alloggio, I, Marroni, F, Ceccobelli, S, Delgado Bermejo, JV, Sarti, FM, Kijas, J, Lenstra, JA, Pilla, F. International Sheep Genomics Consortium. Merino and Merino-derived sheep breeds: a genome-wide intercontinental study. *Genet Sel Evol. 2015 Aug 14;47(1):64.***47**, 64 (2015).

408 Blake, J. A. *et al.* Mouse Genome Database (MGD)-2017: community knowledge resource for the laboratory mouse. *Nucleic Acids Res***45**, D723-D729, doi:10.1093/nar/gkw1040 (2017).

409 Bult, C. J., Eppig, J. T., Blake, J. A., Kadin, J. A. & Richardson, J. E. Mouse genome database 2016. *Nucleic Acids Res***44**, D840-847, doi:10.1093/nar/gkv1211 (2016).

410 Keane, T. M. *et al.* Mouse genomic variation and its effect on phenotypes and gene regulation. *Nature***477**, 289-294, doi:10.1038/nature10413 (2011).

411 Mali, P. *et al.* RNA-guided human genome engineering via Cas9. *Science***339**, 823-826, doi:10.1126/science.1232033 (2013).

412 Wang, H. *et al.* One-step generation of mice carrying mutations in multiple genes by CRISPR/Cas-mediated genome engineering. *Cell***153**, 910-918, doi:10.1016/j.cell.2013.04.025 (2013).

413 Collins, F. S., Rossant, J. & Wurst, W. A mouse for all reasons. *Cell***128**, 9-13, doi:10.1016/j.cell.2006.12.018 (2007).

414 Waterston, R. H. *et al.* Initial sequencing and comparative analysis of the mouse genome. *Nature***420**, 520-562, doi:10.1038/nature01262 (2002).

415 Brown, S. D. & Moore, M. W. The International Mouse Phenotyping Consortium: past and future perspectives on mouse phenotyping. *Mamm Genome***23**, 632-640, doi:10.1007/s00335-012-9427-x (2012).

416 Le Maitre, C., Dahia, CL, Giers, M, Illien-Junger ,S, Cicione, C, Samartzis, D, Vadala, G, Fields, A, Lotz, J. . Development of a standardized histopathology scoring system for human intervertebral disc degeneration: an Orthopaedic Research Society Spine Section Initiative. *JOR Spine***4**, e1167 (2021).

417 Gullbrand, S., Ashinsky, BG, Lai, A, Gansau, J, Crowley, J, Cunha, C, Engiles, JB, Fusellier, M, Muehleman, C, Pelletier, M, Presciutti ,S, Schol ,J, Takeoka, Y, Yurube, T, Zhang, Y, Masuda, K, Iatridis, JC. Development of a standardized histopathology scoring system for intervertebral disc degeneration and regeneration in rabbit models-An initiative of the ORSspine section. *JOR Spine***4**, e1147 (2021).

418 Lai, A., Gansau, J, Gullbrand, SE, Crowley, J, Cunha, C, Dudli, S, Engiles, JB, Fusellier, M, Goncalves, RM, Nakashima, D, Okewunmi, J, Pelletier, M, Presciutti, SM, Schol, J, Takeoka, Y, Yang, S, Yurube, T, Zhang, Y, Iatridis, JC. Development of a standardized histopathology scoring system for intervertebral disc degeneration in rat models: An initiative of the ORS spine section. *JOR Spine***4**, e1150 (2021).
